# Integrating eQTL and GWAS data characterises established and identifies novel migraine risk loci

**DOI:** 10.1007/s00439-023-02568-8

**Published:** 2023-05-28

**Authors:** Ammarah Ghaffar, The International Headache Genetics Consortium, Dale R. Nyholt

**Affiliations:** Statistical and Genomic Epidemiology Laboratory, School of Biomedical Sciences, Faculty of Health, Centre for Genomics and Personalised Health, Queensland University of Technology, Brisbane, QLD 4059 Australia

## Abstract

Migraine—a painful, throbbing headache disorder—is the most common complex brain disorder, yet its molecular mechanisms remain unclear. Genome-wide association studies (GWAS) have proven successful in identifying migraine risk loci; however, much work remains to identify the causal variants and genes. In this paper, we compared three transcriptome-wide association study (TWAS) imputation models—MASHR, elastic net, and SMultiXcan—to characterise established genome-wide significant (GWS) migraine GWAS risk loci, and to identify putative novel migraine risk gene loci. We compared the standard TWAS approach of analysing 49 GTEx tissues with Bonferroni correction for testing all genes present across all tissues (Bonferroni), to TWAS in five tissues estimated to be relevant to migraine, and TWAS with Bonferroni correction that took into account the correlation between eQTLs within each tissue (Bonferroni-matSpD). Elastic net models performed in all 49 GTEx tissues using Bonferroni-matSpD characterised the highest number of established migraine GWAS risk loci (*n* = 20) with GWS TWAS genes having colocalisation (PP4 > 0.5) with an eQTL. SMultiXcan in all 49 GTEx tissues identified the highest number of putative novel migraine risk genes (*n* = 28) with GWS differential expression at 20 non-GWS GWAS loci. Nine of these putative novel migraine risk genes were later found to be at and in linkage disequilibrium with true (GWS) migraine risk loci in a recent, more powerful migraine GWAS. Across all TWAS approaches, a total of 62 putative novel migraine risk genes were identified at 32 independent genomic loci. Of these 32 loci, 21 were true risk loci in the recent, more powerful migraine GWAS. Our results provide important guidance on the selection, use, and utility of imputation-based TWAS approaches to characterise established GWAS risk loci and identify novel risk gene loci.

**Supplementary Information:**

The online version contains supplementary material available at 10.1007/s00439-023-02568-8.

## Introduction

Migraine is a complex neurological trait affecting 14% of the population worldwide (Stovner et al. 2018). According to the Global Burden of Disease Study 2016, migraine is the second leading cause of disability and accounts for more disability than all other neurologic disorders combined (Stovner et al. 2018; Vos et al. 2017). Migraine is characterised by intense, debilitating pain usually on either side of the head. In most cases, migraine is accompanied by nausea, vomiting, numbness, and sensitivity to noise and light (Arnold 2018).

Migraine is a complex polygenic trait and has a strong genetic component with a heritability of 30–60% (Polderman et al. 2015; Sutherland et al. 2019) estimated by different family and twin studies (Honkasalo et al. 1995; Mulder et al. 2003). Genome-wide association studies (GWAS) have substantially improved our understanding of the genetic architecture of migraine and led to the identification of many GWS (*P* < 5 × 10^–8^) single nucleotide polymorphisms (SNPs) associated with migraine (Van Den Maagdenberg et al. 2019). However, as is typical for human complex traits, most of the identified migraine risk SNPs are located in non-coding regions. Therefore, rather than having a direct effect on protein structure and function, these SNPs are believed to act by regulating gene expression. However, identifying which SNP has an effect (i.e., causal SNP) on which gene (causal gene) is not an easy task. In 2016, Gormley et al. published a large migraine GWAS of 59,574 cases and 316,078 controls that identified 44 linkage disequilibrium (LD)-independent ‘index’ SNPs associated with migraine at 38 independent genomic risk loci (Gormley et al. 2016). For convenience, such index SNP loci are typically represented by (annotated with) the nearest gene; however, physical location is not a good proxy for identifying target genes of GWAS SNPs (Visscher et al. 2017) and other factors such as gene density and size complicate the functional interpretation of GWAS risk loci (Van Den Maagdenberg et al. 2019).

Recently, gene-based methods have been developed that leverage GWAS and eQTL data to impute differential expression and test for gene expression associated with the GWAS trait. These methods are termed transcriptome-wide association studies (TWAS). Standard TWAS is usually performed in all available Genotype-Tissue Expression project (GTEx) (Consortium 2015) tissues using Bonferroni correction adjusting for testing all genes present across all tissues (gene-tissue pairs) (Barbeira et al. 2018; Hirbo et al. 2018; Tachmazidou et al. 2019; Torres et al. 2017). Alternatively, TWAS is performed in a single most relevant trait-related tissue with Bonferroni adjustment for the number of genes tested (Feng et al. 2020; Wu et al. 2019). Another group of studies have been published that performed TWAS in a group of trait-relevant tissues with Bonferroni adjustment for the total number of genes tested across the examined tissues (Chen et al. 2021; Guo et al. 2020; Peng et al. 2018). The selection of trait-relevant tissues is based on a literature review or current knowledge of the trait. The approach of selecting tissues is difficult for complex traits such as migraine where the trait-relevant tissues are not known and literature supports multiple hypotheses for the origin of migraine (Mason and Russo 2018). In this paper, we identified tissues related to migraine’s regulatory architecture using genome-wide imputed differential expression enrichment (GIDEE) approach (Ghaffar and Nyholt 2022). Furthermore, we compared three different TWAS methods to (i) characterise established migraine risk loci from Gormley et al. (2016) (Gormley et al. 2016), and (ii) identify GWS differentially expressed genes at putative novel loci (i.e., loci that are not near GWS SNPs in Gormley et al. (2016) migraine GWAS), iii) the putative novel migraine risk genes identified in (ii) were then validated using the recent, more powerful migraine GWAS by Hautakangas et al. (2022) (Hautakangas et al. 2022).

## Materials and methods

**Fig. 1:**
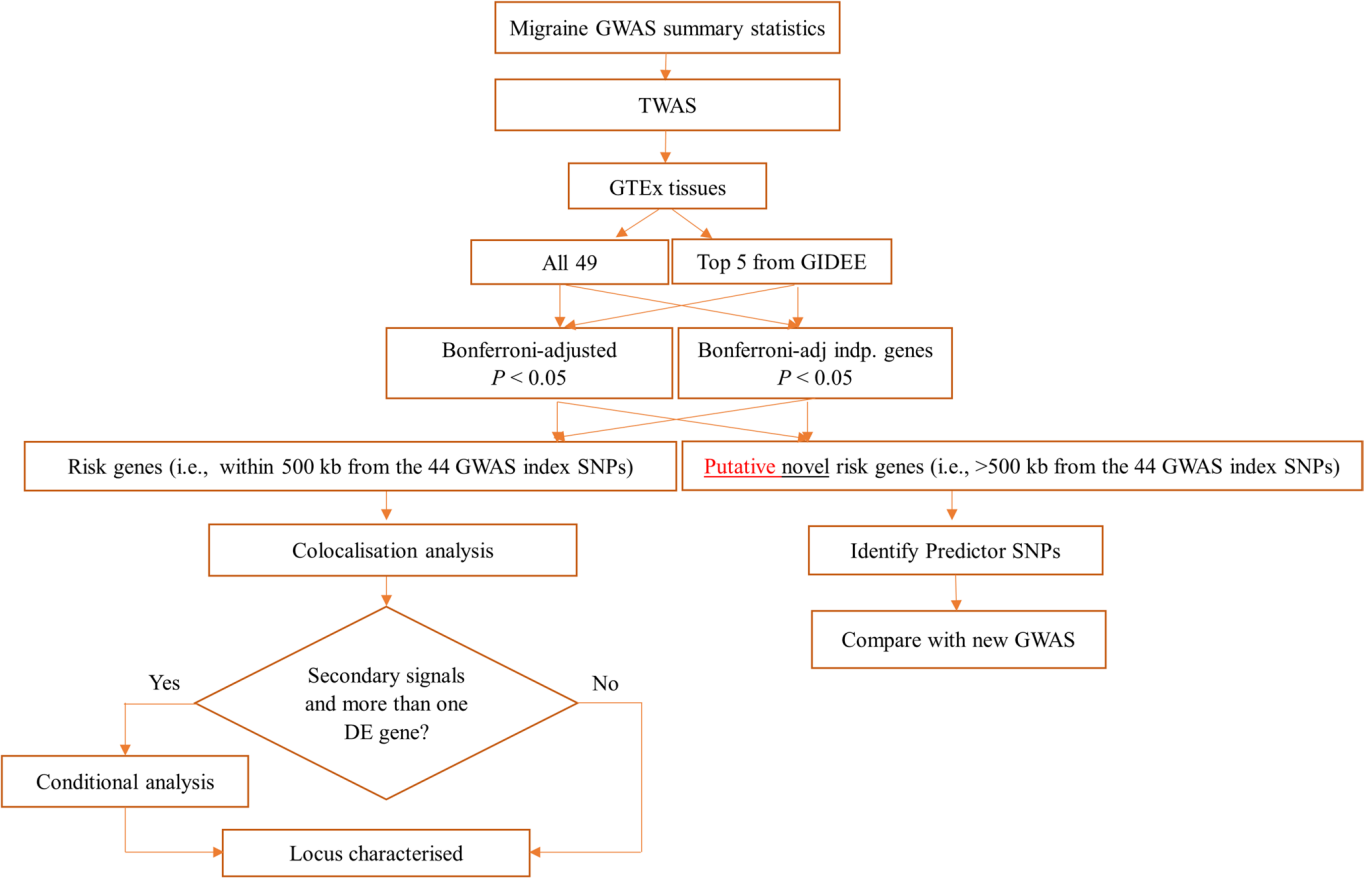
Pipeline followed for characterisation of 44 independent migraine risk SNPs and identification of putative novel risk genes

An overview of the methodology followed in this paper is provided in Fig. 1 and described further in subsequent sections.

### GWAS dataset

The TWAS analyses utilised Gormley et al. (2016) migraine GWAS summary statistics from a GWAS of 59,674 cases and 316,078 controls with European ancestry (Gormley et al. 2016). This study identified 38 independent genomic risk loci containing 44 index SNPs associated with migraine risk (*P* < 5 × 10^–8^). Details of quality control and imputation are provided in the original publication (Gormley et al. 2016). The putative novel migraine risk genes identified by TWAS were validated in the recent, more powerful migraine GWAS by Hautakangas et al. (2022) comprising 102,084 migraine cases and 771,257 controls. The new GWAS dataset contained the Gormley et al. (2016) GWAS and additional GWAS data from 23andMe, Inc. (22,644 cases and 87,729 controls), UK Biobank (10,881 cases and 330,170 controls), GeneRISK (1084 cases and 4857 controls), and HUNT (7801 cases and 32,423 controls) (Hautakangas et al. 2021, 2022). A total of 8,117 GWS (*P* < 5 × 10^–8^) SNPs were identified, of which 170 were LD-independent (*r*^2^ < 0.1) index SNPs. The 170 index SNPs mapped to 123 independent genomic risk loci of which 86 were novel. Details of quality control and imputation are provided in the original publication (Hautakangas et al. 2022).

### Gene expression and eQTL dataset

The majority of GWAS risk SNPs are non-coding and are thus expected to impact the expression of the gene by altering its regulation (Ward and Kellis 2012). eQTL analysis is the most common approach to evaluating the effect of SNPs on gene expression (Grundberg et al. 2012; Morley et al. 2004; Westra et al. 2013). However, eQTL studies are expensive and often limited by the availability of relevant tissue. GTEx provides a resource to address this limitation. The latest version of GTEx hosts data for 54 tissues obtained from 948 donors summing to a total number of 17,382 samples (Consortium 2020). Genotype and eQTL data were available for 49 tissues (N ≥ 70 samples) from 838 donors summing to a total number of 15,201 samples. We downloaded fully processed, filtered, and normalised gene expression matrices (in BED format) for each tissue for GTEx version 8 (v8). The expression and eQTL data used in this project were downloaded from GTEx v8 (https://gtexportal.org/home/).

### Gene expression imputation

The MetaXcan software was used to impute trait-associated differential gene expression in 49 human tissues from GTEx v8. MetaXcan uses a set of reference individuals whose gene expression and genotyping have been measured for the same individuals. The authors of MetaXcan take this information and adjust for sex and experimental/population confounders to impute differential expression in a large independent dataset. GTEx v8 version has two types of prediction models available: MASHR and elastic net models. Both models were used to impute differential expression.

MASHR stands for Multivariate Adaptive Shrinkage in R. In MASHR models, instead of using all SNPs present in the 1 Mb window, the list of SNPs is first shortlisted to only SNPs having a high chance of influencing the expression of that particular gene. This is done by an algorithm DAP-G (Wen et al. 2016), that calculates a posterior inclusion probability (PIP) for each SNP tested, with SNPs having PIP > 0.01 retained for model development(Urbut et al. 2019). Hence, the MASHR models have predictors informed by posterior causal probability that may belong to different LD clusters across tissues, and the effect sizes are based on marginal regression and smoothing across tissues (Barbeira et al. 2020).

Elastic net models use all the SNPs having minor allele frequency > 0.01 present in the 1 Mb window of the gene to determine its effect on the expression of the gene.

The GTEx v8 eQTL data is aligned to the Genome Reference Consortium Human Build 38 (GRCh38, also known as build 38 or hg38) and thus contains variants that were not tested in older GWAS that utilised variants compiled from earlier genome builds. Therefore, the migraine GWAS summary statistics were harmonised and lifted over from build 37 to build 38. Then imputation of summary statistics for missing variants was performed for the migraine GWAS before the differential expression imputation. The gene prediction model SNP weights for each tissue were available in the form of SQLite weight files on predictdb.org (Barbeira et al. 2016) (downloaded from https://predictdb.org/post/2021/07/21/gtex-v8-models-on-eqtl-and-sqtl/).

The elastic net prediction models “elastic_net_eqtl.tar” containing weights of the predictor SNPs on each gene within each tissue along with a single tissue covariance file were retrieved on 11/03/2020. The MASHR models “mashr_eqtl.tar” containing the database and covariance file was retrieved on 01/09/2021.

### Enrichment in migraine-relevant tissues

We used GIDEE to identify tissues enriched with differential gene expression associated with migraine’s regulatory architecture (Ghaffar and Nyholt 2022). GIDEE imputes gene expression and tests for the enrichment of differentially expressed genes in each GTEx tissue. Two methods were used to capture the enrichment of differentially expressed genes; mean squared z-score and empirical Brown’s method adjusted for GTEx tissue sample sizes using a linear regression model lm(enrichment test ~ sample size). Afterwards, the tissues were ranked in ascending order and the average of tissues across both methods was taken. The higher the average rank of tissue, the higher the evidence for differential expression enrichment and the more likely the tissue is pathogenically relevant to migraine. We used the top 5 tissues for the downstream analysis.

### Thresholds to keep type-1 error rate < 0.05 in all tissues and top 5 tissues

Two p value thresholds were used to keep the type 1 error rate < 0.05. First, a strict Bonferroni-adjusted threshold (‘Bonferroni’), which adjusted for the total number of gene-tissue pairs tested (i.e., *p* = 0.05/total number of gene-tissue pairs). The second threshold (Bonferroni-matSpD) utilised a Bonferroni-adjusted threshold, which adjusted for the number of gene-tissue pairs tested after taking into account the substantial covariance in expression across genes within each tissue (i.e., *p* = 0.05/sum of the effective number of independent genes in all tissues). The second threshold was developed because multiple-test adjustment using the total raw number of genes is expected to be too stringent and not reflect true biology (i.e., covariance in gene expression). The effective number of independent genes analysed for differential expression in each tissue was estimated using matrix spectral decomposition (matSpD) (Nyholt 2004). The matSpD approach estimates the effective number of independent variables in a correlation matrix by examining the eigenvalues from spectral decomposition. The expression values for genes whose differential expression was predicted by MetaXcan were extracted from normalised gene expression matrices obtained from GTEx. Briefly, a Pearson correlation matrix was generated using R version 3.6.1 and used as input to the MatSpD.R script downloaded from https://drive.google.com/open?id=1-r-HWsKOD8NfbOG4C4SFIwjj8yYze2Zu. The output was an estimate of the effective number of independent genes along with a p value to efficiently control for type 1 error at 5%.

The above-mentioned multiple testing adjustments were used on two sets of results. One set analysed all 49 GTEx v8 tissues, and another set analysed only the top 5 most likely pathogenically-relevant tissues for migraine.

### Gene expression imputation across tissues

Following the single-tissue MetaXcan analyses, summary-based MultiXcan (SMultiXcan) was used to impute differential gene expression associated with migraine risk using cross-tissue models. SMultiXcan tests the joint effects of gene expression variation across tissue in four steps: (1) gene expression is imputed within single tissues using single tissue elastic net prediction models using models trained on 49 GTEx tissues, (2) a correlation matrix is generated for imputed gene expression and principal components of the predicted expression data matrix are used as explanatory variables, (3) components of smallest variation are discarded from the correlation matrix generated from step 2 to avoid numerical issues caused by collinearity. To select the number of components, the threshold of $\frac{{\lambda }_{max}}{{\lambda }_{i}}<30$ was used, where *λ*_*i*_ is an eigenvalue of the correlation matrix, and (4) joint effects for each gene are estimated using single tissue results from step 1 and the correlation matrix from step 3 to give a joint imputed differential expression for each gene. SMultiXcan provides results for a combined differential expression p value for a single gene, best tissue (i.e., tissue having the lowest differential expression p value), worst tissue (i.e., tissue having the highest differential expression p value), and the mean and standard deviation of z-scores for all tissues in which the specific gene is differentially expressed (Barbeira et al. 2019).

SMultiXcan was applied to all 49 GTEx tissues and on the top 5 most likely pathogenically-relevant tissues for migraine.

### TWAS using 13 GTEx brain tissues

Results from previous analyses by ourselves (Ghaffar and Nyholt 2022; Gormley et al. 2016; Hautakangas et al. 2022) and others (Finucane et al. 2018) did not indicate the 13 GTEx brain tissues were enriched for regulatory signals at migraine GWAS loci and/or GWAS loci were enriched for genes specifically expressed in GTEx brain tissues. However, given migraine is a neurological disorder that affects the brain, for completeness and the interests of readers, we also performed analogous TWAS analyses restricted to the 13 GTEx brain tissues.

### Co-localisation analysis

COLOC (Giambartolomei et al. 2014) was used to assess the probability that the same SNP (effect) associated with migraine risk also influences gene expression. COLOC utilises Bayesian statistics to test for all possible combinations of association with both datasets (migraine GWAS and eQTL) at the SNP level. This requires setting prior probabilities for three combinations: P_1_, P_2_ and P_12_. P_1_ and P_2_ are set to 1 × 10^–4^ and refer to the association of the SNP in dataset 1 and dataset 2, respectively. P_12_ is set to 1 × 10^–5^ and refers to the prior probability of association of the SNP in both datasets. In other words, P_12_ defines the prior probability that given the SNP is associated in dataset 1, what is the probability that the SNP is associated in dataset 2. Setting P_12_ as 1 × 10^–5^ means that 1 out of 10 SNPs associated with dataset 1 is also associated with dataset 2. Co-localisation analysis enables the separation of the LD contaminated (i.e., correlated neighbouring) genes by calculating posterior probabilities (PP) to distinguish between pleiotropy (i.e., the same SNP influencing both datasets) and LD. For each gene that was differentially expressed and that passed multiple testing for each threshold, eQTL summary statistics were extracted for cis-SNPs from GTEx v8 and tested for co-localisation with migraine-risk SNPs. Given the default prior probabilities of a SNP’s association with expression (P_1_ = 10^−4^), trait (P_2_ = 10^−4^) and both (P_12_ = 10^−5^), COLOC produces posterior probabilities for five hypotheses—H0: no association with either trait; H1: association with trait 1, not with trait 2; H2: association with trait 2, not with trait 1; H3: association with trait 1 and trait 2, two independent SNPs; and H4: association with trait 1 and trait 2, one shared SNP. A large posterior probability for H3 (PP3) means that there are two independent SNPs associated with each trait at the same locus. A large posterior probability for H4 (PP4) means there is a single SNP associated with both traits at a given locus. Therefore, the higher the PP4 (PP4 > 0.5), the higher the confidence for co-localisation (pleiotropy), where the same SNP that affects migraine, affects the expression of the gene. Co-localisation may also be inferred via a low PP3 (PP3 < 0.5).

### Loci characterisation

Significant differentially expressed genes with high PP4 or low PP3 were assigned to a migraine risk locus if they were within ± 500 kb of a GWS index SNP from Gormley et al. (2016). For significant differentially expressed genes that were >500 kb away from the migraine index SNPs located on chromosomes 1–22 from Gormley et al. (2016), we hypothesised that these genes were putative novel migraine risk genes (i.e., because they were not at GWS GWAS risk loci). Loci having LD-independent secondary GWAS signals and more than two differentially expressed genes were subjected to conditional analysis for both signals (SNPs) at the given locus.

### Conditional association analysis

Genomic loci with secondary GWAS signals (secondary index SNPs) and more than one differentially expressed gene were subjected to conditional analyses to determine the marginal effect of each GWAS index SNP on gene expression. At these genomic loci, conditional association analysis was performed separately for each index SNP using GCTA-COJO (Yang et al. 2012). GCTA-COJO performs approximate conditional association analysis using GWA summary statistics and LD estimated from a reference population. Each index SNP was given as an input to GCTA-COJO with a window size of 2 MB. The association p values of all SNPs within 2 MB of index SNPs were re-calculated/conditioned. The TWAS pipeline (pre-processing, harmonisation and imputation) was then re-run on each set of conditioned GWAS summary statistics. The 1000 Genomes Project Phase 3 European population reference panel, downloaded from https://ctg.cncr.nl/software/magma (de Leeuw et al. 2015), was provided as a reference panel to estimate LD. The results of the conditional analysis were visualised via LocusZoom plots (Pruim et al. 2010).

### Validation of putative novel risk genes and loci in a new GWAS

The putative novel migraine risk genes identified by TWAS were checked to see if they were within ± 500 kb of the 170 index SNPs and implicated by TWAS in the recent more powerful migraine GWAS by Hautakangas et al. (2022) (Hautakangas et al. 2021). Predictor SNPs from the putative novel migraine risk genes [from TWAS analysis of the Gormley et al. (2016) data] were also tested for LD with the 170 migraine index SNPs.

### Independent gene-based test

To test whether the putative novel migraine risk genes identified by TWAS were more likely to be located at GWS risk loci in a recent, more powerful migraine GWAS we performed a novel gene-based analysis. First, we performed gene-based GATES (Li et al. 2011) test implemented in the Fast Association Tests (FAST) (Chanda et al. 2013) using the Gormley et al., (2016) migraine GWAS summary statistics. GATES integrates association evidence (p values) of SNPs assigned to a gene to obtain an overall p value for the association of the entire gene. The flanking region for each gene was increased to 500 kb to match the flanking region used in our analysis to define a putative novel locus. The output includes the most significant SNP (‘topSNP’) assigned to a gene, which we used to represent the gene. We note that neighbouring genes may have correlated results due to LD between the topSNP assigned to each gene. To estimate the effective number of independent genes, we estimated the effective number of independent topSNPs using the genetic type 1 error calculator (GEC) (Li et al. 2012). The topSNPs identified by GATES genes were given as input. GEC divides the input SNPs into LD blocks, assuming that these blocks are independent by ensuring that the SNPs between the blocks are not in LD (*r*^2^ < 0.1) utilising 1000 Genome Project European reference genotype data. Given the list of SNPs, GEC calculates the effective number of independent SNPs that were later used in binomial tests.

## Results

### Top 5 migraine-relevant tissues

**Fig. 2:**
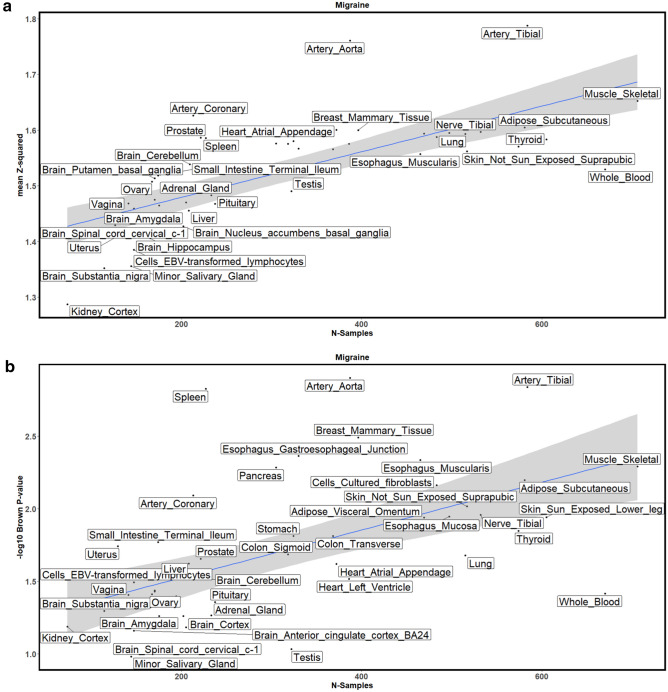
Enrichment methods result for the migraine GWAS. **A** Shows the mean squared z-score enrichment test results; **B** shows the Brown’s p value enrichment test results

Figure 2 shows the mean squared z-score and empirical Brown’s method p value for the migraine GWAS adjusted for GTEx tissues sample size. Figure 2A shows the linear regression plot of the mean squared z-score against the GTEx sample size for all tissues. The blue line shows the best fit through the data. Artery Aorta is the furthest tissue from the fitted line, thus implying that it had the highest enrichment of differentially expressed genes (as represented by z-score). Figure 2B shows that Artery Aorta had the highest enrichment of genes differentially expressed (as represented by Brown’s p value). GTEx tissue sample sizes and ranking based on the enrichment tests are provided in Supplementary Table 1. Afterwards, we used the average of the tissue ranks from the two enrichment tests to select the top 5 tissues for downstream analysis. The top 5 tissues enriched with differentially expressed genes were the artery aorta, artery tibial, spleen, artery coronary, and pancreas (Ghaffar and Nyholt 2022). The GIDEE prioritisation of artery aorta, artery tibial and artery coronary tissues, firmly corresponds with published data that show migraine GWAS loci are strongly associated with vascular tissues (Finucane et al. 2018; Gormley et al. 2016; Hautakangas et al. 2022); while the prioritisation of spleen and pancreas tissues is supported by a 2017 study that suggested migraine is associated with general, not nervous-system-specific, inflammatory processes (Wang et al. 2017)—i.e., spleen and pancreas are involved in inflammation via their respective roles in the immune and endocrine system (Hiller-Sturmhöfel & Bartke 1998; Mota & Madden 2022).

### Significance thresholds across approaches

We compared 10 approaches that used different prediction models (elastic net, SMultiXcan, or MASHR), different significance thresholds (Bonferroni or Bonferroni-matSpD), and different sets of tissues (all 49 GTEx tissues or top 5 tissues). Table 1 lists the total number of genes present in all tissues whose differential expression is imputed (using elastic net models). The effective number of independent genes within each tissue was estimated using the matSpD approach. A considerable difference was observed in multiple test burdens for the raw number of genes (MTB_r_) and the effective number of independent genes (MTB_e_). As described in the methods section, SMultiXcan imputes gene expression considering the correlation of eQTLs across multiple tissues and generates a single p value. Given differences in gene–gene co-expression correlation across tissues, it is not feasible to estimate the effective number of independent genes via the matSpD approach, therefore multiple test correction for the SMultiXcan results used a Bonferroni adjustment for the total raw number of genes tested.

**Table 1:** Summary of the total number of genes and effective number of independent genes present in each tissue using the elastic net and SMultiXcan prediction models for all 49 GTEx tissues and the top 5 tissues

Tissues	Elastic net			
	All 49		Top 5	
	MTB_r_	MTB_e_	MTB_r_	MTB_e_
Adipose subcutaneous	8645	5037		
Adipose visceral omentum	7338	3985		
Adrenal gland	4841	2584		
Artery aorta	7598	4321	7598	4321
Artery coronary	4042	2198	4042	2198
Artery tibial	8611	5016	8611	5016
Brain amygdala	2786	1458		
Brain anterior cingulate cortex BA24	3543	1724		
Brain caudate basal ganglia	5001	2500		
Brain cerebellar hemisphere	5752	3114		
Brain cerebellum	6793	3807		
Brain cortex	5499	2758		
Brain frontal cortex BA9	4560	2188		
Brain hippocampus	3687	1864		
Brain hypothalamus	3650	1946		
Brain nucleus accumbens basal ganglia	4850	2490		
Brain putamen basal ganglia	4433	2171		
Brain spinal cord cervical c-1	3249	1713		
Brain substantia nigra	2557	1338		
Breast mammary tissue	6460	3368		
Cells cultured fibroblasts	8931	4245		
Cells EBV-transformed lymphocytes	2903	1562		
Colon sigmoid	6165	3425		
Colon transverse	6303	2603		
Esophagus gastroesophageal junction	6288	3410		
Esophagus mucosa	8517	4689		
Esophagus muscularis	8225	4375		
Heart atrial appendage	6638	3591		
Heart left ventricle	6011	2769		
Kidney cortex	1641	852		
Liver	3768	1924		
Lung	7965	4380		
Minor salivary gland	2914	1529		
Muscle skeletal	7581	4250		
Nerve tibial	10,007	5840		
Ovary	3587	1950		
Pancreas	5895	3383	5895	3383
Pituitary	5685	3131		
Prostate	4300	2282		
Skin not sun exposed suprapubic	8648	4974		
Skin sun exposed lower leg	9297	5202		
Small intestine terminal ileum	3668	1667		
Spleen	5770	3231	5770	3231
Stomach	5150	2375		
Testis	9973	3444		
Thyroid	9649	5715		
Uterus	2537	1331		
Vagina	2559	1159		
Whole blood	7252	3426		
Total number of genes	281,722	148,316	31,916	18,151
p value threshold	1.77 × 10^–7^	3.37 × 10^–7^	1.56 × 10^–6^	2.75 × 10^–6^

*MTB*_*r*_ multiple test burden for the raw number of genes_,_ *MTB*_*e*_ multiple test burden for the effective number of genes, *All 49* all 49 tissues present in GTEx version 8, *Top 5* top 5 GTEx tissues associated with migraine

The total number of genes tested across all 49 GTEx tissues by SMultiXcan was 21,646, therefore the Bonferroni-adjusted significance threshold was 2.30 × 10^–6^ (0.05/21,646). Similarly, the total number of genes tested across the top 5 GIDEE tissues was 12,988, resulting in a Bonferroni-adjusted significance threshold was 3.84 × 10^–6^. As shown in Table 1, the multiple test burden considerably differs across the six approaches, with a trend of decreasing burden from left to right. Table 2 shows the number of genes present in each tissue using MASHR prediction models and multiple test burden across the different approaches.

**Table 2:** Summary of the total number of genes and effective number of independent genes present in each tissue using MASHR prediction models for all 49 GTEx tissues and top 5 tissues

Tissues	MASHR			
	All 49		Top 5	
	MTB_r_	MTB_e_	MTB_r_	MTB_e_
Adipose subcutaneous	14,233	8388		
Adipose visceral omentum	14,096	7780		
Adrenal gland	13,025	7154		
Artery aorta	13,855	7949	13,855	7949
Artery coronary	13,289	7438	13,289	7438
Artery tibial	14,006	8215	14,006	8215
Brain amygdala	12,186	6617		
Brain anterior cingulate cortex BA24	12,875	6245		
Brain caudate basal ganglia	13,533	6858		
Brain cerebellar hemisphere	13,160	7276		
Brain cerebellum	13,406	7605		
Brain cortex	13,647	6937		
Brain frontal cortex BA9	13,477	6527		
Brain hippocampus	12,929	6691		
Brain hypothalamus	13,107	7125		
Brain nucleus accumbens basal ganglia	13,443	7053		
Brain putamen basal ganglia	13,110	6614		
Brain spinal cord cervical c-1	12,451	6837		
Brain substantia nigra	11,978	6457		
Breast mammary tissue	14,067	7387		
Cells cultured fibroblasts	13,536	6399		
Cells EBV-transformed lymphocytes	11,818	6508		
Colon sigmoid	13,779	7799		
Colon transverse	14,016	5557		
Esophagus gastroesophageal junction	13,730	7571		
Esophagus mucosa	14,073	7794		
Esophagus muscularis	14,095	7595		
Heart atrial appendage	13,493	7482		
Heart left ventricle	12,681	5874		
Kidney cortex	10,530	5836		
Liver	12,142	6399		
Lung	14,473	8077		
Minor salivary gland	13,271	7247		
Muscle skeletal	12,924	7342		
Nerve tibial	14,912	8785		
Ovary	13,102	7440		
Pancreas	13,149	7632	13,149	7632
Pituitary	14,047	7868		
Prostate	13,779	7561		
Skin not sun exposed suprapubic	14,412	8379		
Skin sun exposed lower leg	14,697	8329		
Small intestine terminal ileum	13,422	6109		
Spleen	13,517	7693	13,517	7693
Stomach	13,499	6076		
Testis	17,119	6150		
Thyroid	14,832	8871		
Uterus	12,535	6853		
Vagina	12,271	5585		
Whole blood	12,160	5768		
Total number of genes	657,887	349,757	54,299	38,929
p value threshold	7.60 × 10^–8^	1.42 × 10^–7^	9.21 × 10^–7^	1.28 × 10^–6^

*MTB*_*r*_ multiple test burden for the raw number of genes_,_ *MTB*_*e*_ multiple test burden for the effective number of genes, *All 49* all 49 tissues present in GTEx version 8, *Top 5* top 5 GTEx tissues associated with migraine as identified

Although not part of our primary analyses, we also performed TWAS analyses restricted to the 13 GTEx brain tissues. The MTB_r_ and MTB_e_ for elastic net models were 8.87 × 10^–7^ (0.05/56360) and 1.72 × 10^–6^ (0.05/29071), respectively. Similarly, the MTB_r_ and MTB_e_ for MASHR models were 2.95 × 10^–7^ (0.05/169302) and 5.63 × 10^–7^ (0.05/88842), respectively. The total number of genes tested across the 13 GTEx brain tissues by SMultiXcan was 14,218, therefore the Bonferroni-adjusted significance threshold was 3.52 × 10^–6^ (0.05/14,218).

MetaXcan outputs a z-score that quantifies the association of predicted differential gene expression with the trait. The positive or negative sign of the z-score indicates overexpression or underexpression of the gene’s association with migraine, respectively. A two-sided p value is calculated from the z-score. For each approach mentioned in Tables 1 and 2, genes having a p value less than the respective approach’s p value threshold are considered significant.

### Comparison across approaches

For each TWAS approach, the genes crossing the respective multiple test burden that were present within ± 500 kb of a GWS independent index SNP in the Gormley et al. 2016 migraine GWAS were identified. Table 3 shows the total number of genes and the number of genes present within ± 500 kb of an established migraine index SNP.

**Table 3:** Number of genes passing multiple test burden and number of genes present in the vicinity of migraine index SNPs and number of independent genomic loci

TWAS model	Tissues	Threshold	Genes	Genes within ± 500 kb of a migraine index SNP (genomic loci)
Elastic Net	Top 5	Bonferroni	43 (31)	28 (18)
		Bonferroni matSpD	47 (33)	29 (18)
	All 49	Bonferroni	52 (29)	35 (20)
		Bonferroni matSpD	61 (34)	40 (22)
	13 Brain	Bonferroni	21 (15)	12 (7)
		Bonferroni matSpD	25 (18)	14 (8)
SMultiXcan	Top 5	Bonferroni	47 (32)	31 (18)
	All 49	Bonferroni	67 (38)	39 (18)
	13 Brain	Bonferroni	28 (20)	15 (9)
MASHR	Top 5	Bonferroni	36 (21)	27 (15)
		Bonferroni matSpD	49 (27)	30 (16)
	All 49	Bonferroni	36 (22)	30 (17)
		Bonferroni matSpD	42 (25)	34 (19)
	13 Brain	Bonferroni	23 (15)	18 (11)
		Bonferroni matSpD	32 (20)	24 (13)

Table 3 shows that elastic net with Bonferroni-matSpD threshold for all 49 tissues identified the highest number of differentially expressed genes within ± 500 kb of a migraine index SNP, i.e., 40 genes present at 22 independent loci. SMultiXcan identified 31 and 39 genes for the top 5 and all 49 tissues, at 18 independent loci, respectively. Whereas, MASHR identified 27 and 30 genes, at 15 and 16 independent loci, for the top 5 tissues using Bonferroni and Bonferroni-matSpD, respectively. Using all 49 tissues MASHR identified 30 and 34 genes, at 17 and 19 independent loci, respectively. Overall, across the three TWAS models, our primary analyses of all 49 tissues and the top 5 tissues, identified a total of 128 genes at 58 independent loci, of which 66 genes at 26 loci were within ± 500 kb of a migraine index SNP.

Compared to using the top 5 tissues and all 49 GTEx tissues, TWAS using the 13 GTEx brain tissues identified considerably fewer differentially expressed genes across the genome and in the vicinity of migraine index SNPs (Table 3 and Supplementary Table 2a and 2b). Across the three TWAS models, analysis of the 13 brain tissue subgroup identified a total of 55 genes at 31 independent loci, of which 45 genes at 23 loci overlapped with the 128 genes identified in the primary analyses. In contrast, only 10 genes (*RP11-326G21.1*, *NEURL3*, *GOT1*, *RBM20*, *GPR26*, *CDIP1*, *FGF11*, *ATP5SL*, *TMEM91*, *TGFB1*) at 8 independent loci identified in the brain subgroup analyses were not identified in the primary analyses (Supplementary Table 2c). Given these 10 genes were not as significant as the genes implicated by the all 49 tissue analyses, and the 13 GTEx brain tissues were not previously found to be enriched for regulatory signals at migraine GWAS loci, we consider these ten genes to be less robust than the genes associated in our primary TWAS analyses. Therefore, we only present results in the main text from subsequent analyses for the top 5 and all 49 GTEx tissues.

Gormley et al. (2016) identified 44 GWS index SNPs; however, because the TWAS approaches only impute gene expression for genes on chromosomes 1 to 22 and one of the 44 index SNPs (rs12845494) was on chromosome X, only 43 index SNP loci could be characterised by TWAS. Out of these 43 index SNP loci, 30 (25 independent) loci had evidence of differentially expressed genes from at least one of the 10 approaches mentioned in Tables 1 and 2. Table 4 presents a list of 30 index SNPs and genes characterised by each approach along with the genes that are nearest to the index SNP. Given LD between the migraine index SNPs and eQTL SNPs used to impute differential expression could produce false-positive results in the form of coincidental differential expression, significant differentially expressed genes were further examined using co-localisation analysis by COLOC (Giambartolomei et al. 2014). Genes having PP3 < 0.5 against a migraine index SNP are shown in Table 4. Genes in bold show more robust co-localised signals (PP4 > 0.5) for at least one of the tissues in which the gene is expressed.

**Table 4:** A comprehensive gene list characterised at each locus based on MTB and co-localised signals

RsIDs	Chr	Pos	Nearest gene	EN-All-B	EN-All-M	EN-Top5-B	EN-Top5-M	SM-All	SM-Top5	MR-All-B	MR-All-M	MR-Top5-B	MR-Top5-M
rs2078371	1	115,677,183	*TSPAN2*	***TSPAN2***^a^	***TSPAN2***^a^	***TSPAN2***^a^	***TSPAN2***^a^	***NGF*** ***TSPAN2***^a^	***TSPAN2***^a^	***TSPAN2***^a^	***NGF*** ***TSPAN2***^a^	***NGF*** ***TSPAN2***^a^	***NGF*** ***TSPAN2***^a^
rs7544256	1	115,824,398	*TSPAN2*	***TSPAN2***^a^	***TSPAN2***^a^	***TSPAN2***^a^	***TSPAN2***^a^	***NGF*** ***TSPAN2***^a^	***TSPAN2***^a^ *VANGL1*	***TSPAN2***^a^	***NGF*** ***TSPAN2***^a^	***NGF*** ***TSPAN2***^a^	***NGF*** ***TSPAN2***^a^
rs6693567	1	150,510,660	*ADAMTSL4*	***ADAMTSL4***^a^ ***ECM1***^a^ ***MRPS21*** ***NBPF19*** ***TARS2*** *ANXA9*	***ADAMTSL4***^a^ ***ECM1***^a^ ***MRPS21*** ***NBPF19*** ***TARS2*** *ANXA9* ***PLEKHO1***	***ADAMTSL4***^a^ ***ECM1***^a^	***ADAMTSL4***^a^ ***ECM1***^a^	***ADAMTSL4***^a^ ***ECM1***^a^ *MCL1* ***MRPS21*** *NBPF19* ***PLEKHO1*** ***TARS2***	***ADAMTSL4***^a^ ***ECM1***^a^	***ADAMTSL4***^a^ ***ECM1***^a^ ***MRPS21*** ***TARS2***	***ADAMTSL4***^a^ ***ECM1***^a^ ***MRPS21*** ***TARS2***	***ADAMTSL4***^a^ ***ECM1***^a^ ***RPRD2***	***ADAMTSL4***^a^ ***ECM1***^a^ ***MRPS21*** ***RPRD2***
rs1925950	1	156,450,740	*MEF2D*	***MEF2D***^a^	***MEF2D***^a^	***MEF2D***^a^	***MEF2D***^a^	***MEF2D***^a^	***MEF2D***^a^	***MEF2D***^a^	***MEF2D*** ^a^	***MEF2D***^a^	***MEF2D***^a^
rs138556413	2	203,832,867	*CARF*	***CARF*** ***ICA1L*** ***NBEAL1***	***CARF*** ***FAM117B*** ***ICA1L*** ***NBEAL1*** ***WDR12***	***FAM117B*** ***ICA1L*** ***NBEAL1***	***FAM117B*** ***ICA1L*** ***NBEAL1***	*NA*	***FAM117B*** ***ICA1L*** ***NBEAL1***	NA	*ICA1L* ***WDR12***	***ICA1L*** ***NBEAL1***	***ICA1L*** ***NBEAL1***
rs566529	2	234,756,811	*TRPM8*	NA	NA	NA	NA	NA	NA	*HJURP*	*HJURP*	NA	NA
rs10166942	2	234,825,093	*TRPM8*	NA	NA	NA	NA	NA	NA	*HJURP*	*HJURP*	NA	NA
rs7684253	4	57,727,311	*SPINK2*	***REST***	***REST***	NA	NA	NA	NA	***REST***	***NOA1*** ***REST***	NA	NA
rs9349379	6	12,903,957	PHACTR1	***PHACTR1***^a^	***PHACTR1***^a^	***PHACTR1***^a^	***PHACTR1***^a^	***PHACTR1***^a^	***PHACTR1***^a^	***PHACTR1***^a^	***PHACTR1***^a^	***PHACTR1***^a^	***PHACTR1***^a^
rs10456100	6	39,183,470	KCNK5	NA	NA	NA	NA	NA	NA	NA	***KCNK5***	***KCNK5***	***KCNK5***
rs4839827	6	96,853,967	FHL5	***FHL5***^a^ ***UFL1***^a^	***FHL5***^a^ ***UFL1***^a^	***FHL5***^a^ ***UFL1***^a^	***FHL5***^a^ ***UFL1***^a^	***FHL5***^a^ ***UFL1***^a^	***FHL5***^a^ ***UFL1***^a^	***FHL5***^a^ ***UFL1***^a^	***FHL5***^a^ ***UFL1***^a^	***FHL5***^a^ ***UFL1***^a^	***FHL5***^a^ ***UFL1***^a^
rs67338227	6	97,042,147	FHL5	***FHL5***^a^ ***UFL1***^a^	***FHL5***^a^ ***UFL1***^a^	***FHL5***^a^ ***UFL1***^a^	***FHL5***^a^ ***UFL1***^a^	***FHL5***^a^ ***UFL1***^a^	***FHL5***^a^ ***UFL1***^a^	***FHL5***^a^ ***UFL1***^a^	***FHL5***^a^ ***UFL1***^a^	***FHL5***^a^ ***UFL1***^a^	***FHL5***^a^ ***UFL1***^a^
rs28455731	6	121,846,038	GJA1	***GJA1***	***GJA1***	***GJA1***	***GJA1***	***GJA1***	***GJA1***	NA	NA	NA	NA
rs1268083	6	126,049,040	*HEY2*	***HEY2***^a^	***HEY2***^a^	***HEY2***^a^	***HEY2***^a^	***HEY2***^a^	***HEY2***^a^	***HEY2*** ^a^ ***NCOA7***	***HEY2*** ^a^ ***NCOA7***	***HEY2***^a^	***HEY2***^a^
rs186166891	7	40,406,876	*SUGCT*	***SUGCT***	***SUGCT***	***SUGCT***	***SUGCT***	***SUGCT***	***SUGCT***	NA	NA	NA	NA
rs6478241	9	119,252,629	ASTN2	NA	NA	NA	NA	NA	NA	*TRIM32*	*TRIM32*	NA	NA
rs10786156	10	96,014,622	*PLCE1*	***NOC3L***	***NOC3L***	NA	NA	***PLCE1***	NA	***PLCE1***	***PLCE1***	NA	NA
rs75473620	10	96,019,029	*PLCE1*	***NOC3L***	***NOC3L***	NA	NA	***PLCE1***	NA	***PLCE1***	***PLCE1***	NA	NA
rs12260159	10	100,702,737	*HPSE2*	***HPSE2***	***HPSE2***	***HPSE2***	***HPSE2***	NA	***HPSE2***	***HPSE2***	***HPSE2***	***HPSE2***	***HPSE2***
rs2223089	10	124,210,160	ARMS2	NA	NA	NA	NA	***ARMS2*** ***HTRA1***	NA	*HTRA1*	*HTRA1*	*HTRA1*	*HTRA1*
rs4910165	11	10,674,044	*MRVI1*	NA	NA	NA	NA	NA	NA	***LYVE1***	***LYVE1***	NA	NA
rs10895275	11	102,083,608	*YAP1*	***RP11-732A21.2***	***RP11-732A21.2***	***RP11-732A21.2***	***RP11-732A21.2***	NA	***RP11-732A21.2***	NA	NA	***RP11-732A21.3*** *YAP1*	***RP11-732A21.3*** *YAP1*
rs1024905	12	4,518,140	*FGF6*	***C12orf4***^a^	***C12orf4***^a^	***C12orf4***^a^ ***CCND2***	***C12orf4***^a^ ***CCND2***	***C12orf4***^a^ ***CCND2***	***C12orf4***^a^ ***CCND2***	***C12orf4***^a^	***C12orf4***^a^	***C12orf4***^a^ ***CCND2***	***C12orf4***^a^ ***CCND2***
rs11172055	12	57,308,260	*LRP1*	***LRP1***^a^ *NEMP1* ***STAT6***	***LRP1***^a^ *NEMP1* ***STAT6***	***LRP1***^a^ *NEMP1*	***LRP1***^a^ *NEMP1*	***LRP1***^a^ ***STAT6*** *HSD17B6*	***LRP1***^a^ *NEMP1*	***LRP1***^a^ ***STAT6***	***LRP1***^a^ ***STAT6***	***LRP1***^a^ ***STAT6***	***LRP1***^a^ ***STAT6***
rs11172113	12	57,527,283	*LRP1*	***LRP1***^a^ *NEMP1* ***STAT6*** *B4GALNT1*	***LRP1***^a^ *NEMP1* ***STAT6*** *B4GALNT1*	***LRP1***^a^ *NEMP1*	***LRP1***^a^ *NEMP1*	***LRP1***^a^ ***STAT6*** *HSD17B6* *B4GALNT1*	***LRP1***^a^ *NEMP1*	***LRP1***^a^ ***STAT6***	***LRP1***^a^ ***STAT6***	***LRP1***^a^ ***STAT6***	***LRP1***^a^ ***STAT6***
rs11624776	14	93,595,591	*ITPK1*	***BTBD7***	***BTBD7***	***BTBD7*** ***ITPK1***	***BTBD7*** ***ITPK1***	***BTBD7***	***BTBD7*** ***ITPK1***	NA	NA	NA	NA
rs77505915	16	75,442,143	*CFDP1*	***BCAR1***^a^ ***CFDP1*** ***TMEM170A***	***BCAR1***^a^ ***CFDP1*** ***TMEM170A***	***BCAR1***^a^	***BCAR1***^a^	***BCAR1***^a^ ***CFDP1*** ***TMEM170A***	***BCAR1***^a^	***BCAR1***^a^ ***CFDP1*** ***TMEM170A*** *RP11-77K12.9*	***BCAR1***^a^ ***CFDP1*** ***TMEM170A*** *RP11-77K12.9*	***BCAR1***^a^ *RP11-77K12.9*	***BCAR1***^a^ *RP11-77K12.9*
rs4081947	16	87,579,870	*ZCCHC14*	NA	***ZCCHC14***	***ZCCHC14***	***ZCCHC14***	NA	***ZCCHC14***	NA	NA	NA	NA
rs17857135	17	78,262,161	*RNF213*	***RNF213***	***RNF213***	NA	NA	***RNF213***	NA	***RNF213*** *SLC26A11*	***RNF213*** *SLC26A11*	NA	NA
rs4814864	20	19,469,817	*SLC24A3*	***SLC24A3***	***SLC24A3***	***SLC24A3***	***SLC24A3***	***SLC24A3***	***SLC24A3***	NA	NA	NA	NA

*EN-All-B* elastic net all 49 tissues using Bonferroni correction, *EN-All-M* elastic net all 49 tissues using Bonferroni matSpD correction, *EN-top-B* elastic net top 5 tissues using Bonferroni correction, *EN-Top-M* elastic net top 5 tissues using Bonferroni matSpD correction, *SM-All* SMultiXcan all 49 tissues using Bonferroni correction, *SM-top* SMultiXcan top 5 tissues using Bonferroni correction, *MR-All-B* MASHR all 49 tissues using Bonferroni correction, *MR-All-M* MASHR all 49 tissues using Bonferroni matSpD correction, *MR-top-B* MASHR top 5 tissues using Bonferroni correction, *MR-top-M* MASHR top 5 tissues using Bonferroni matSpD correction

^a^Represent common genes among all approaches. Genes in bold represent the more robust co-localised signals (PP4 > 0.5) observed in at least one tissue

A total of 12 loci (migraine index SNP rs2078371, rs7544256, rs6693567, rs1925950, rs9349379, rs4839827**,** rs67338227, rs1268083, rs1024905, rs11172055, rs11172113, and rs77505915) had a common set of genes implicated by all approaches. Among these 12 loci, three loci have secondary association signals (rs7544256 near rs2078371, rs67338227 near rs4839827, and rs11172113 near rs11172055). It is important to note that NA in Table 4 refers to the fact that there might be a gene at that locus differentially expressed but a co-localisation signal was not present (i.e., PP3 < 0.5 or PP4 > 0.5).

Supplementary Table 3 contains genes differentially expressed at each index SNP along with the TWAS p value and COLOC PP3 and PP4 values for all elastic net, SMultiXcan, and MASHR models (as in Table 4).

There were five loci (rs28455731, rs186166891, rs11624776, rs4081947, and rs4814864), that had differentially expressed and co-localised genes (having a co-localised signal for eQTL and migraine risk) identified by elastic net models but not by MASHR models. Table 5 shows genes prioritised at these migraine risk loci using elastic net models and respective TWAS p values using MASHR models in the same tissues.

**Table 5:** List of genes and loci implicated via the elastic net but not by MASHR models

rsIDs	Chr	Pos	Gene	Tissue	enet-p	MASHR-p
rs28455731	6	121,846,038	*GJA1*	Artery tibial	5.67 × 10^–9^	1.36 × 10^–5^
rs186166891	7	40,406,876	*SUGCT*	Artery aorta Artery tibial	2.92 × 10^–15^ 4.05 × 10^–12^	Gene not present Gene not present
rs11624776	14	93,595,591	*BTBD7* *ITPK1*	Artery aorta Artery_tibial	2.46 × 10^–8^ 6.23 × 10^–7^	0.460 0.007
rs4081947	16	87,579,870	*ZCCHC14*	Artery_tibial	3.12 × 10^–7^	5.31 × 10^–6^
rs4814864	20	19,469,817	*SLC24A3*	Artery aorta Artery tibial	1.75 × 10^–16^ 1.04 × 10^–17^	0.401 0.267

*Chr* chromosome, *Pos* position of index SNP, *Enet-p* TWAS p value using elastic net prediction model for respective tissue, *MASHR-p* TWAS p value using MASHR prediction model for respective tissue

Table 5 shows that all genes except *SUGCT* implicated via elastic net were also implicated via MASHR models. *GJA1* and *ZCCHC14* did not reach the relevant significance level threshold of 1.28 × 10^–6^ for MASHR models using matSpD in the top 5 tissues (Table 2). Two genes implicated at rs11624776 (*BTBD7* and *ITPK1*) were not significant for the MASHR models. This probably reflects differences in the predictor SNPs used in MASHR models compared to the elastic net models (e.g., the MASHR predictor SNPs for these genes capture less of the variation in genetically regulated gene expression). Similarly, *SLC24A3* at rs4814864 was not significant using the MASHR model.

Five loci were implicated with MASHR prediction models but not with elastic net models (Table 6). Most of the genes implicated with MASHR did not have predicted gene expression with elastic net models in tissues where a significant association was found. However, it is important to note that out of five loci, three loci were characterised based solely on low co-localisation PP3 values (as opposed to low PP3 and high PP4 values). Two genes *KCNK5* and *LVYE1* had PP4 > 0.5.

**Table 6:** List of genes and loci implicated via MASHR but not by elastic net models

rsIDs	Chr	Pos	Gene	Tissue	MASHR-p	enet-p
rs566529	2	234,756,811	*HJURP*	Nerve_tibial Ovary Skin_not_sun_exposed_suprapubic Small_intestine_terminal_ileum testis	1.15 × 10^–8^ 6.93 × 10^–8^ 1.25 × 10^–8^ 1.33 × 10^–8^ 8.75 × 10^–8^	Gene not present Gene not present 0.206593362 Gene not present 4.23 × 10^–7^
rs10166942	2	234,825,093	*HJURP*	Nerve_tibial Ovary Skin_not_sun_exposed_suprapubic Small_intestine_terminal_ileum testis	1.15 × 10^–8^ 6.93 × 10^–8^ 1.25 × 10^–8^ 1.33 × 10^–8^ 8.75 × 10^–8^	Gene not present Gene not present 0.206593362 Gene not present 4.23 × 10^–7^
rs10456100	6	39,183,470	*KCNK5*	Artery_tibial Breast_mammary_tissue	7.97 × 10^–8^ 7.97 × 10^–8^	Gene not present 9.21 × 10^–7^
rs6478241	9	119,252,629	*TRIM32*	Brain_nucleus_accumbens_basal_ganglia Brain_spinal_cord_cervical_c-1 Brain_substantia_nigra Minor_salivary_gland	3.18 × 10^–8^ 3.15 × 10^–8^ 5.70 × 10^–8^ 1.11 × 10^–7^	Gene not present Gene not present Gene not present Gene not present
rs4910165	11	10,674,044	*LYVE1*	Brain_cerebellum	6.45 × 10^–12^	Gene not present

*Chr* chromosome, *Pos* position of index SNP, *MASHR-p* TWAS p value using MASHR prediction model for respective tissue, *Enet-p* TWAS p value using elastic net prediction model for respective tissue

There were eight loci (rs138556413, rs7684253, rs10786156, rs75473620, rs12260159, rs10895275, rs17857135, and rs2223089) that did not have a uniform set of genes across all approaches. For these loci, we suggest that the gene(s) most significantly associated (differentially expressed) with migraine is most likely the causal gene. For example, of the five genes (*CARF*, *ICAIL*, *NBEAL1*, *FAM117B*, and *WDR12*) at the rs138556413 locus, *ICAIL* and *NBEAL1* have the strongest TWAS p value with migraine as compared to other genes. Therefore, *ICAIL* and *NBEAL1* are the most probable causal genes at rs138556413. *REST* is prioritised at locus rs7684253. However, *REST* was not identified in the top 5 migraine-relevant tissues. *HPSE2* is the most likely causal gene at rs12260159. *RP11-732A21.2* is the most likely causal gene at rs10895275. *RNF213* and *HTRA1* are hypothesised as causal genes at rs17857135 and rs2223089, respectively. Interestingly, near the rs10786156 index SNP (GWAS *P* = 2.0 × 10^–14^), there is a secondary index SNP rs75473620 (GWAS *P* = 5.8 × 10^–9^), and the different TWAS models prioritised different genes at this locus. Elastic net models prioritised *NOC3L*, while MASHR and SMultiXcan prioritised *PLCE1*. *NOC3L* is differentially expressed in the brain nucleus accumbens basal ganglia with a p value of 4.27 × 10^–8^. Whereas *PLCE1* is differentially expressed in the brain cerebellar hemisphere with a p value of 5.33 × 10^–9^. Therefore, *PLCE1* is hypothesised to be the most likely causal gene at this locus. A summary of the number of loci characterised by each of the 10 approaches (as in Table 4) is shown in Table 7.

**Table 7:** Overall comparison of all 10 approaches based upon COLOC

Approach	No. of genes (PP3 < 0.5) (indp. genomic loci)	No. of genes (PP4 > 0.5) (indp. genomic loci)
EN-All-B	32 (19)	29 (19)
EN-All-M	39 (20)	33 (20)
EN-Top5-B	24 (17)	23 (17)
EN-Top5-M	24 (17)	23 (17)
SM-All	31 (16)	27 (16)
SM-Top5	25 (17)	23 (17)
MR-All-B	27 (17)	12 (14)
MR-All-M	32 (19)	26 (16)
MR-Top5-B	23 (14)	20 (13)
MR-Top5-M	24 (14)	21 (13)

*EN-All-B* elastic net all 49 tissues using Bonferroni correction, *EN-All-M* elastic net all 49 tissues using Bonferroni matSpD correction, *EN-top-B* elastic net top 5 tissues using Bonferroni correction, *EN-Top-M* elastic net top 5 tissues using Bonferroni matSpD correction, *SM-All* SMultiXcan all 49 tissues using Bonferroni correction, *SM-top* SMultiXcan top 5 tissues using Bonferroni correction, *MR-All-B* MASHR all 49 tissues using Bonferroni correction, *MR-All-M* MASHR all 49 tissues using Bonferroni matSpD correction, *MR-top-B* MASHR top 5 tissues using Bonferroni correction, *MR-top-M* MASHR top 5 tissues using Bonferroni matSpD correction

Table 7 shows that elastic net prediction models prioritised robust genes that have strong evidence of co-localisation of GWAS and eQTL signals (i.e., all loci with a PP3 < 0.5 also had a PP4 > 0.5). In terms of prioritising the highest number of loci characterised, elastic net single tissue models that take into account correlation among genes (EN-All-M) performed best characterising 20 migraine risk loci all with robust co-localisation (PP3 < 0.5 and PP4 > 0.5).

### Conditional analysis

**Fig. 3:**
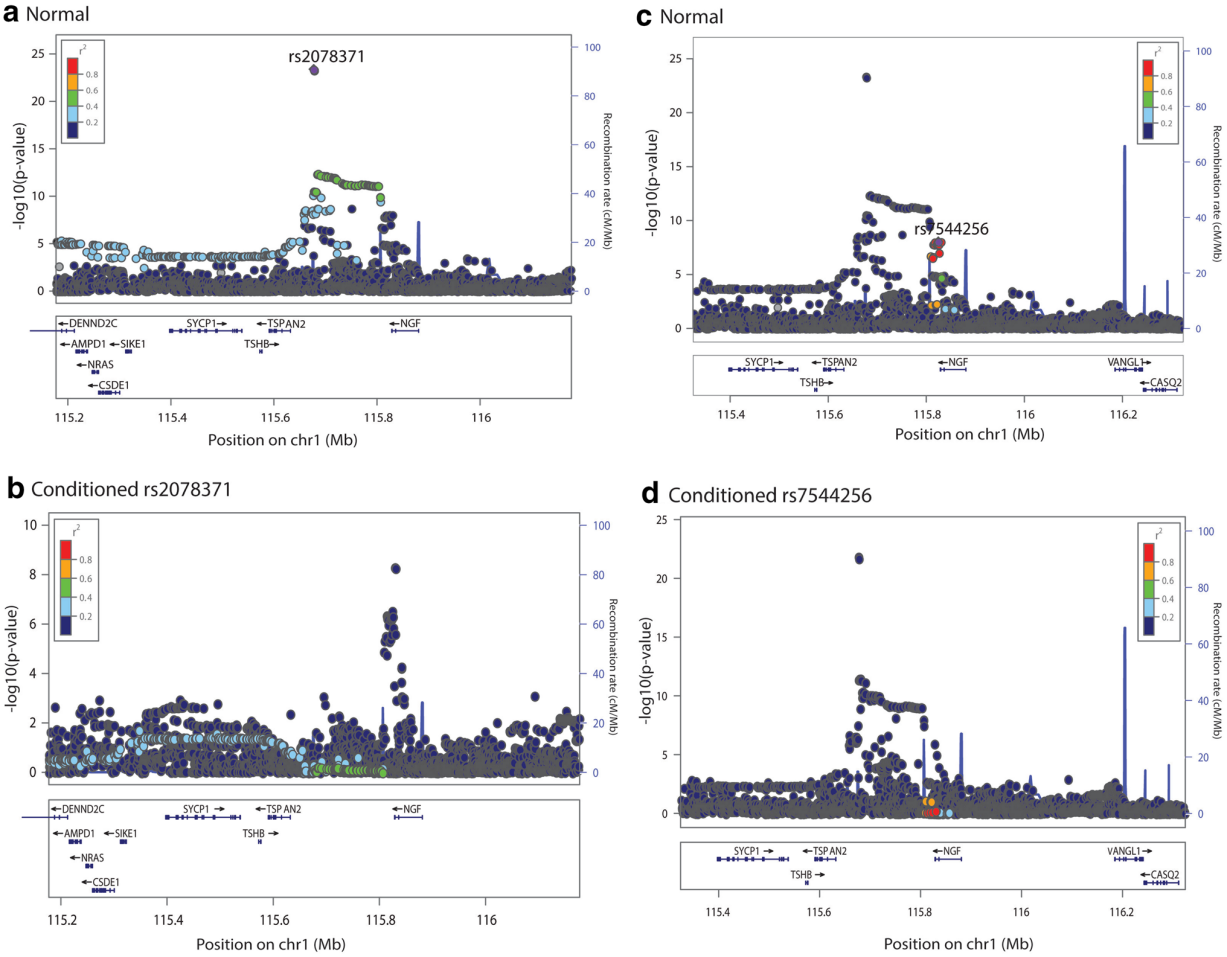
Conditional analysis for the locus containing index SNPs rs2078371 and rs7544256 and more than two DE genes crossing multiple testing burden. Normal refers to unconditioned/original migraine GWAS summary statistics

**Fig. 4:**
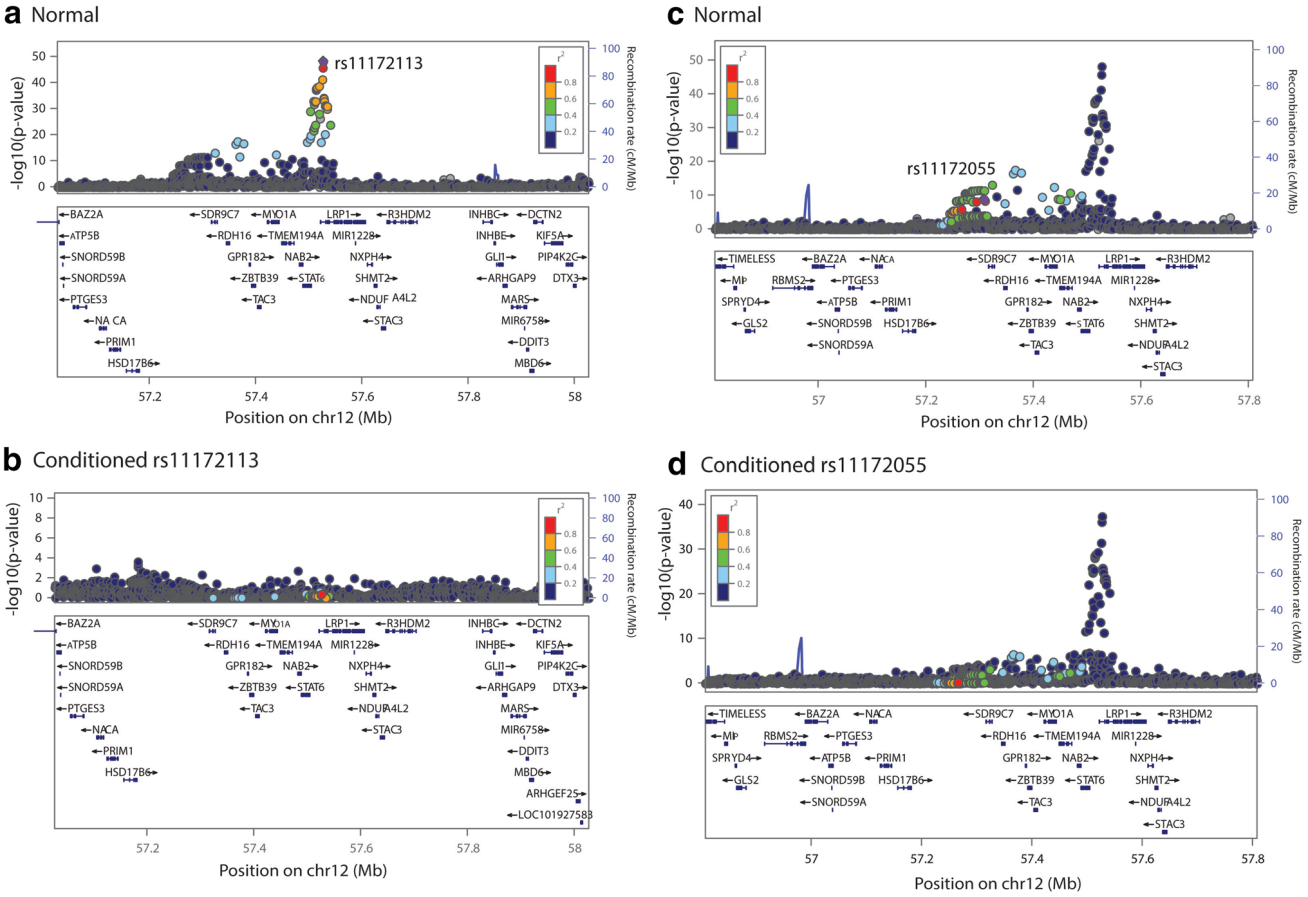
Conditional analysis for the locus containing index SNPs rs11172113 and rs11172055 and more than two DE genes crossing multiple testing burden. Normal refers to unconditioned/original migraine GWAS summary statistics

**Fig. 5:**
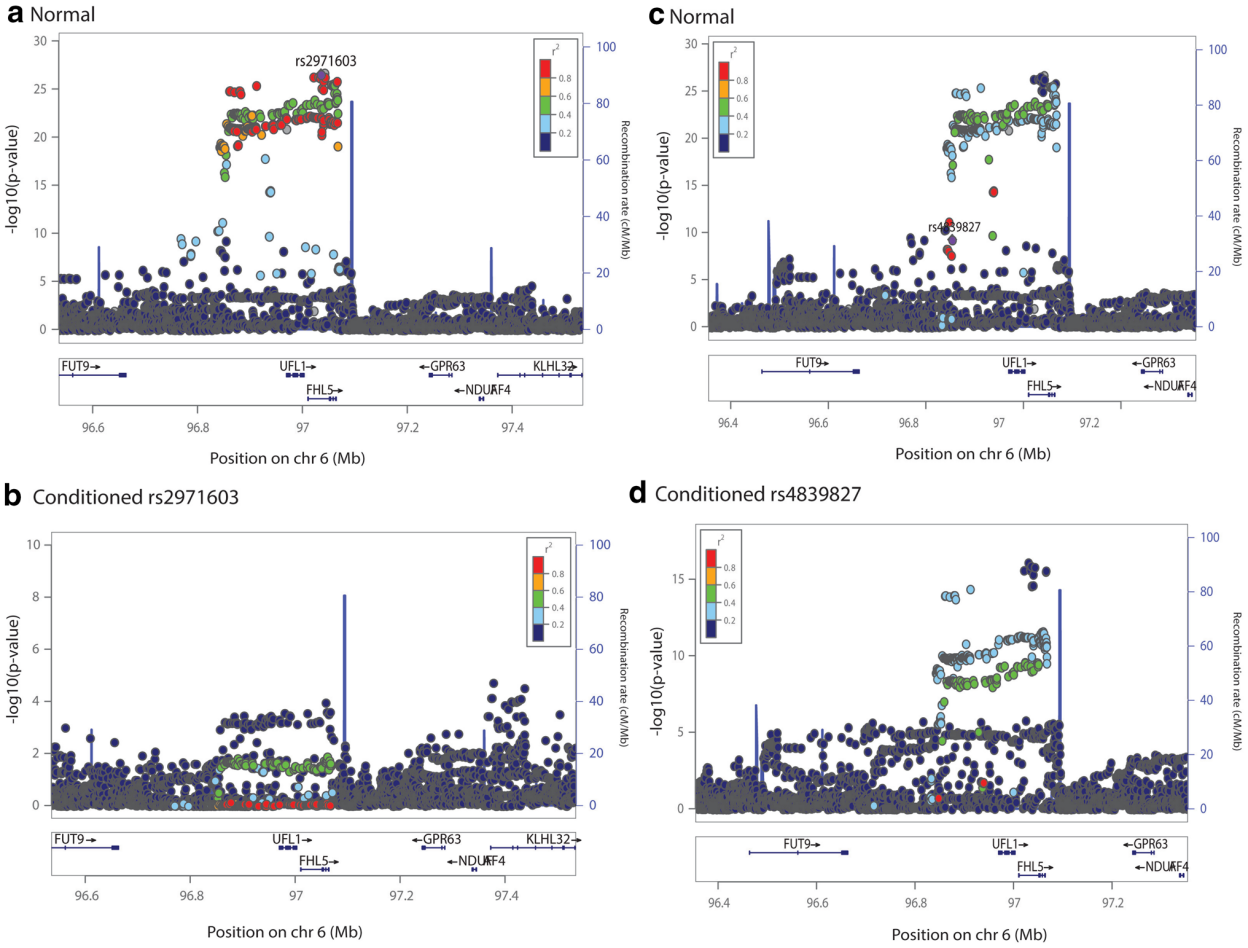
Conditional analysis for the locus containing index SNPs rs2971603 (a proxy for rs67338227) and rs4839827 and more than two DE genes crossing multiple testing burden. Normal refers to unconditioned/original migraine GWAS summary statistics

There were three genomic loci (rs2078371/rs7544256, rs11172055/rs11172113, and rs4839827/rs67338227) having independent migraine index SNPs and more than one gene differentially expressed. *TSPAN2* and *NGF* were in 1 Mb window of rs2078371/rs7544256, *LRP1* and *STAT6* were in the 1 Mb window of rs11172055/rs11172113, and *FHL5* and *UFL1* were in the 1 Mb window of rs67338227/rs4839827. To examine and identify which index SNP affects the differential expression of which gene, conditional analyses were performed for each index SNP at these loci using GCTA-COJO. We note that, rs67338227 was not present in the 1000 Genomes Project LD reference, therefore, a proxy SNP rs2971603 located 6,729 bp upstream of rs67338227 having a similar effect allele frequency (0.227), beta (0.087) and p value (2.83 × 10^–27^) was used. The conditioning window was set to 2 Mb around the index SNP. The TWAS pipeline was re-run including harmonisation, summary statistics imputation, and differential expression imputation (using MASHR models) using the conditioned GWAS summary statistics. Table 8, Figs. 3, 4, and 5 show the conditioned results.

**Table 8:** TWAS results before and after conditioning on index SNPs

Gene	Chr	Tissue	TWAS *P*		
			Normal	Conditioned	
				rs2078371	rs7544256
*TSPAN2*	1	Artery aorta	1.95 × 10^–13^	0.674126887	1.85 × 10^–12^
*NGF*	1	Artery aorta	9.35 × 10^–8^	0.839511484	2.55 × 10^–11^
				rs11172113	rs11172055
*LRP1*	12	Artery aorta	5.64 × 10^–49^	0.926949555	3.72 × 10^–38^
		Artery coronary	1.57 × 10^–46^	0.382734997	6.46 × 10^–37^
		Artery tibial	5.64 × 10^–49^	0.926949555	3.72 × 10^–38^
*STAT6*	12	Artery aorta	8.07 × 10^–40^	0.613315511	1.60 × 10^–29^
		Artery coronary	3.02 × 10^–40^	0.585206809	5.04 × 10^–30^
		Spleen	5.69 × 10^–43^	0.340679136	4.26 × 10^–34^
				rs2971603	rs4839827
*FHL5*	6	Artery aorta	5.25 × 10^–22^	0.784582007	1.92 × 10^–11^
		Artery tibial	5.74 × 10^–23^	0.925143001	4.74 × 10^–12^
*UFL1*	6	Artery coronary	2.07 × 10^–20^	0.585072206	6.60 × 10^–13^
		Artery tibial	9.14 × 10^–21^	0.534423271	1.28 × 10^–12^
		Pancreas	6.35 × 10^–17^	0.350427160	3.91 × 10^–8^

Figures 3, 4 and 5 show LocusZoom plots for normal and conditioned GWAS on each primary and secondary index SNP present at each locus (± 500 kb). The x axis of the LocusZoom plot shows the bp position on the chromosome. The y-axis shows the GWAS p value for each SNP. The bottom panel of the LocusZoom plot shows the name and position of genes derived from the UCSC browser (Karolchik et al. 2003). The LD between SNPs is displayed as *r*^2^ and is colour-coded according to the strength of correlation. The European 1000 Genomes Project data was used as a reference panel to calculate LD. LocusZoom plots also display local recombination hotspots as well. The recombination rate is displayed on the right side of the y axis.

Conditional analysis results show that rs2078371 is driving the differential expression results for both *TSPAN2* and *NGF*, as when conditioned on rs2078371, the differential expression p values for these genes drop from 1.95 × 10^–13^ to 0.674 and 9.35 × 10^–8^ to 0.839, respectively (i.e., they were no longer significantly differentially expressed with migraine). In contrast, when conditioned on rs7544256, the TWAS results for *TSPAN2* and *NGF* remained significant. Similarly, the conditional analyses showed that rs11172113 is driving differential expression for *LRP1* and *STAT6*. Conditioning on rs11172113 resulted in decreased TWAS significance in all tissues. Whereas both genes *LRP1* and *STAT6* remained significant after conditioning on rs11172055. The same pattern of results was observed for rs2971603 (rs67338227) and rs4839827. The primary lead SNP rs2971603 (rs67338227) was driving the differential expression for *FHL5* and *UFL1*.

In an attempt to further characterise these migraine risk loci, we attempted to identify genes with imputed differential expression driven by the secondary index SNPs (rs7544256, rs11172055, and 4,839,827) by comparing the ‘normal’ TWAS p value and conditioned TWAS p value for all genes present at these loci. Supplementary Table 4 and Supplementary Table 5 show the list of top genes present ± 500 kb of each index SNP having the smallest p value when conditioned on the secondary SNP, along with normal TWAS p value. However, no genes at these loci were identified to have differential expression driven by the secondary SNP. Thus, indicating that the primary lead SNPs are driving differential expression of both genes at these loci.

### Characterisation of putative novel loci

Across all the approaches, a total of 62 putative novel migraine risk genes were identified. The total number of genes from each threshold (Tables 1, 2) not within ± 500 kb of the 43 genome-wide index SNPs and within ± 500 kb of 170 index SNPs is shown in Table 9.

**Table 9:** Total number of significant differentially expressed genes and number of genes not within ± 500 kb of the 43 migraine index SNPs from Gormley et al. (2016) and within ± 500 kb of 170 index SNPs from Hautakangas et al. (2022)

Model	Tissues	Threshold	Total genes (genomic loci)	Genes not within ± 500 kb of the 43 index SNPs (genomic loci)	Genes within ± 500 kb of 170 index SNPs (genomic loci)
Elastic Net	Top 5	Bonferroni	43 (31)	15 (13)	12 (11)
		Bonferroni matSpD	47 (33)	18 (15)	13 (11)
	All 49	Bonferroni	52 (29)	17 (9)	11 (5)
		Bonferroni matSpD	61 (34)	21 (12)	14 (7)
SMultiXcan	Top 5	Bonferroni	47 (32)	16 (14)	10 (9)
	All 49	Bonferroni	67 (38)	28 (20)	20 (14)
MASHR	Top 5	Bonferroni	36 (21)	9 (6)	9 (6)
		Bonferroni matSpD	49 (27)	19 (11)	18 (10)
	All 49	Bonferroni	36 (22)	6 (5)	5 (4)
		Bonferroni matSpD	42 (25)	8 (6)	7 (5)

Table 9 shows that SMultiXcan analysis using all 49 tissues identified the highest number of genes (28) that were not present within ± 500 kb of the GWS migraine index SNP loci from Gormley et al. (2016). Out of these 28 genes, 20 genes were present at 14 loci from Hautakangas et al. (2022). We consider these genes to be putative novel migraine risk genes. Among the single tissue models, the elastic net models identified a higher number of genes compared to the MASHR models. Moreover, more loci were identified using the elastic net models for the top 5 migraine-related tissues (11) as compared to the MASHR models.

Next, we determined how many of the putative novel migraine risk genes identified from TWAS analysis of the Gormley et al. (2016) GWAS data, were located near a GWS locus in the Hautakangas et al. (2022) migraine GWAS. The Hautakangas et al. (2022) migraine GWAS also performed TWAS using MetaXcan MASHR prediction models followed by COLOC and another approach named FOCUS (Mancuso et al. 2019). FOCUS is a probabilistic/Bayesian approach that models the correlation among genes identified from TWAS and calculates a posterior probability for each gene within a region being a causal gene (Mancuso et al. 2019).

In terms of loci, a total of 24 loci having differentially expressed genes with at least one of the TWAS approaches, were identified using Gormley et al. (2016) migraine GWAS data. Table 10 shows the list of all putative novel migraine risk genes that were present within ± 500 kb of the Hautakangas et al. (2022) migraine GWAS index SNPs for the 10 TWAS approaches. This table also indicates whether the gene was verified by TWAS (i.e., FOCUS and/or MetaXcan and COLOC) in Hautakangas et al. (2022).

**Table 10:** Putative novel differentially expressed (migraine risk) gene loci from TWAS analysis of Gormley et al. (2016) data identified and/or verified by Hautakangas et al. (2022)

rsIDs	Chr	Pos	EN-All-B	EN-All-M	EN-Top5-B	EN-Top5-M	SM-All	SM-Top5	MR-All-B	MR-All-M	MR-Top5-B	MR-Top5-M
rs2124663	1	2,833,427	*TNFRSF14*	*TNFRSF14*			*TNFRSF14*					
rs61561984	1	15,490,865					*TMEM51*^*a*^					
rs12057629	1	15,538,493					*TMEM51*^*a*^					
rs1472662	1	39,590,409	*MACF1*^*a*^* BMP8A*^*a*^* PABPC4*^*a*^	*MACF1*^*a*^* BMP8A*^*a*^* PABPC4*^*a*^	*BMP8A*^*a*^	*BMP8A*^*a*^* PABPC4*^*a*^	*MACF1*^*a*^	*BMP8A*^*a*^* PABPC4*^*a*^				
rs68002561	1	149,880,863	*HIST2H2BF* *BOLA1*^*a*^* SV2A*^*a*^	*HIST2H2BF BOLA1*^*a*^ *SV2A*^*a*^	*SV2A*^*a*^	*SV2A*^*a*^	*RP11-353N4.6 FCGR1A HIST2H2BF BOLA1*^*a*^	*SV2A*^*a*^			*SF3B4*^*a*^	*SV2A*^*a*^ *SF3B4*^*a*^
rs7544531	1	149,897,217	*HIST2H2BF BOLA1*^*a*^* SV2A*^*a*^	*HIST2H2BF BOLA1*^*a*^ *SV2A*^*a*^	*SV2A*^*a*^	*SV2A*^*a*^	*RP11-353N4.6 FCGR1A HIST2H2BF BOLA1*^*a*^	*SV2A*^*a*^			*SF3B4*^*a*^	*SV2A*^*a*^ *SF3B4*^*a*^
rs56140113	1	206,843,108					*EIF2D*	*EIF2D*	*MAPKAPK2*^*a*^	*MAPKAPK2*^*a*^		
rs4907224	2	96,576,609			*TMEM127*^*ab*^	*TMEM127*^*ab*^	*ITPRIPL1*^*ab*^					
rs72923449	2	176,978,383										*HOXD8*
rs1499963	3	124,607,055			*ITGB5*^*ab*^	*ITGB5*^*ab*^		*ITGB5*^*ab*^				
rs42854	5	74,963,277					*POC5*^*a*^					
rs6556059	5	172,645,766		*NKX2-5*^*ab*^			*NKX2-5*^*ab*^					
rs9468830	6	30,749,712					*HCG20*					
rs74434374	6	31,850,308		*ABHD16A*	*ABHD16A*	*ABHD16A*	*ABHD16A*	*ABHD16A*				
rs7932866	11	46,548,094	*MDK*^*ab*^ *ATG13*^*ab*^ *LRP4*	*MDK*^*ab*^ *ATG13*^*ab*^ *LRP4*	*MDK*^*ab*^ *LRP4*	*MDK*^*ab*^ *LRP4*	*LRP4*	*LRP4*			*CHRM4*^*b*^	*CHRM4*^*b*^* HARBI1*^*a*^* ATG13*^*ab*^
rs12419507	11	47,669,619			*CELF1*	*CELF1*					*FNBP4*^*a*^	*FNBP4*^*a*^
rs28756401	14	58,761,912			*LINC00216*^*ab*^	*LINC00216*^*ab*^		*LINC00216*^*ab*^				*ARID4A*^*ab*^
rs12598836	16	4,534,482			*HMOX2*^*ab*^	*HMOX2*^*ab*^	*NMRAL1*^*ab*^* UBALD1*	*HMOX2*^*ab*^	*DNAJA3*^*ab*^* HMOX2*^*ab*^	*DNAJA3*^*ab*^* NMRAL1*^*ab*^* HMOX2*^*ab*^	*HMOX2*^*ab*^	*HMOX2*^*ab*^
rs9894634	17	1,967,501										*RP11667K14.3*^*a*^
rs74182632	19	19,406,126					*TM6SF2 GATAD2A*^*a*^* YJEFN3*		*MAU2*^*a*^	*MAU2*^*a*^	*MAU2*^*a*^	*SUGP1* *MAU2*^*a*^
rs1982072	19	41,864,509	*EXOSC5*^*a*^	*B9D2*^*ab*^ *EXOSC5*^*a*^					*B9D2*^*ab*^	*B9D2*^*ab*^		*B9D2*^*ab*^
rs3092262	20	45,580,290			*EYA2*^*a*^	*EYA2*^*a*^						
rs910187	20	45,841,052			*EYA2*^*a*^	*EYA2*^*a*^						
rs28451064	21	35,593,827			*LINC00310*^*b*^	*LINC00310*^*b*^	*LINC00310*^*b*^	*LINC00310*^*b*^		*KCNE2*^*ab*^	*MRPS6*^*b*^* SLC5A3*^*ab*^* AP000318.2*^*b*^* KCNE2*^*ab*^	*MRPS6*^*b*^* SLC5A3*^*ab*^* LINC00310*^*b*^* AP000318.2*^*b*^* KCNE2*^*ab*^

*EN-All-B* elastic net all 49 tissues using Bonferroni correction, *EN-All-M* elastic net all 49 tissues using Bonferroni matSpD correction, *EN-top-B* elastic net top 5 tissues using Bonferroni correction, *EN-Top-M* elastic net top 5 tissues using Bonferroni matSpD correction, *SM-All* SMultiXcan all 49 tissues using Bonferroni correction, *SM-top* SMultiXcan top 5 tissues using Bonferroni correction, *MR-All-B* MASHR all 49 tissues using Bonferroni correction, *MR-All-M* MASHR all 49 tissues using Bonferroni matSpD correction, *MR-top-B* MASHR top 5 tissues using Bonferroni correction, *MR-top-M* MASHR top 5 tissues using Bonferroni matSpD correction

^a^Represents genes verified by FOCUS in Hautakangas et al. (2022)

^b^Represents genes verified by MetaXcan and COLOC in Hautakangas et al. (2022)

Afterwards, we examined the 62 putative novel risk genes identified by TWAS and assigned a topSNP (from Gormley et al. 2016) to each gene as described in the methods section. We first identified the largest topSNP p value from the 62 putative novel genes (RNF41, *P*_*max*_ = 0.000923), and then identified all the genes across the genome that had an equivalent level of significance with a topSNP p value (5 × 10^–8^ < topSNP *P* < *P*_*max*_). We then compared the topSNP p values from Gormley et al. (2016) to Hautakangas et al. (2022). Given the topSNPs may not be independent, we estimated the effective number of independent topSNPs by examining their LD relationship using GEC. The proportion of the effective number of independent topSNPs of the putative novel migraine risk genes that had a topSNP with Hautakangas *P* < 5 × 10^–8^ was 21/32 = 0.656, while the proportion of the effective number of independent topSNPs of all the genes that had a topSNP with Hautakangas *P* < 5 × 10^–8^ was 105/2140 = 0.049. Compared to the empirically derived expected proportion of 0.049, a significantly higher proportion (0.6563) of the 62 putative novel migraine risk genes mapped to GWS migraine risk loci in Hautakangas et al (2022) (one-sided binomial test *P* = 2.38 × 10^–20^).

The majority of the differentially expressed genes identified by our TWAS analysis of the Gormley et al. (2016) migraine GWAS were verified in Hautakangas et al. (2022) migraine GWAS. It is important to note that Hautakangas assigned genes to a particular locus using a 1 Mb window upstream and a 1 Mb window downstream. However, we used a window of 500 kb upstream and 500 kb downstream of a gene to assign it to a particular locus. One gene, *CYP2C9* was identified using our analysis and verified by Hautakangas et al. (2022), but it was not within ± 500 kb of a GWAS index SNP. In Hautakangas et al. (2022), *CYP2C9* was assigned to the rs2274224 SNP locus at chr10:96,039,597 (build 37), which is 658,818 bp upstream from the *CYP2C9* transcription start site (chr10:96,698,415).

Importantly, all the genes verified by Hautakangas et al. (2022) were implicated via our TWAS and co-localisation. That is, MetaXcan and co-localisation probability (PP4 > 0.5) that the same SNP affecting gene expression is affecting migraine risk. Similarly, genes implicated by FOCUS analysis had a posterior inclusion probability (PIP) > 0.5 being a causal gene. It is also worth noting that the significance threshold used by the Hautakangas et al. (2022) migraine TWAS analysis utilised a conservative Bonferroni adjusted threshold, which adjusted for the total number of gene-tissue pairs tested (i.e., *p* = 0.05/total number of gene-tissue pairs).

Table 11 contains some putative novel migraine risk genes that were present within ± 500 kb of Hautakangas et al. (2022) index SNPs but were not verified by their MetaXcan and FOCUS analyses. For those genes, we extracted the TWAS predictor SNP having the smallest GWAS p value from the Gormley et al. (2016) migraine GWAS dataset and tested it for LD with the lead index SNPs from Hautakangas et al. (2022) and also checked the GWAS p value for the predictor SNP in Hautakangas et al. (2022). Table 11 shows that although these 14 genes were not found to be differentially expressed in the Hautakangas et al. (2022) TWAS analyses, nine genes had predictor SNPs that were in LD (*r*^2^ > 0.1) with Hautakangas et al. (2022) index SNPs. The predictor SNPs for *TNFRSF14* (*r*^2^ = 0.0003), *HIST2H2BF* (*r*^2^ = 0.0004), *FCGR1A* (*r*^2^ = 0.0004), and *YJEFN3* (*r*^2^ = 0.0087) were not in LD with Hautakangas et al. (2022) index SNPs. This may indicate the presence of secondary GWAS association signals at these loci which were not detected due to insufficient power, but which contributed to our observed TWAS association. Table 11 also shows that as the sample size increased in the Hautakangas et al. (2022) GWAS, the majority of the predictor SNPs became more significant. Supplementary Tables 6–11 list the LD between predictor SNPs for all genes and index SNPs from Hautakangas using MASHR, elastic net and SMultiXcan prediction models.

**Table 11:** Association and LD for putative novel migraine risk gene loci predictor SNPs in Hautakangas et al. (2022)

Gene (tissue)	Chr	Predictor SNP	GWAS p value for predictor SNP in Gormley et al. (2016)	GWAS p value for predictor SNP in Hautakangas et al. (2022)	Lead index SNP in Hautakangas et al. (2022)	LD between predictor and lead index SNP	GWAS p value for lead SNP in Hautakangas et al. (2022)
*HOXD8* (artery tibial)	2	rs114763776	1.97 × 10^–5^	0.062848	rs72923449	0.2979	4.66 × 10^–8^
*TNFRSF14* (small intestine terminal)	1	rs868718	3.89 × 10^–17^	1.57 × 10^–36^	rs2124663	0.0003	2.44 × 10^–9^
*HIST2H2BF* (esophagus muscularis)	1	rs698915	4.62 × 10^–7^	3.47 × 10^–11^	rs68002561	0.0004	3.61 × 10^–10^
*RP11-353N4.6* (Lung)	1	rs1046332	1.76 × 10^–6^	1.31 × 10^–7^	rs68002561	0.7383	3.61 × 10^–10^
*FCGR1A* (whole blood)	1	rs698915	4.62 × 10^–7^	3.47 × 10^–11^	rs68002561	0.0004	3.61 × 10^–10^
*EIF2D* (spleen)	1	rs6658181	1.45 × 10^–5^	7.49 × 10^–5^	rs56140113	0.0159	7.76 × 10^–9^
*EIF2D* (thyroid)		rs4072677	1.06 × 10^–5^	5.83 × 10^–6^	rs56140113	0.2291	7.76 × 10^–9^
*ABHD16A* (pancreas)	6	rs1802127	6.25 × 10^–6^	4.16 × 10^–6^	rs74434374	0.1828	4.51 × 10^–9^
*HCG20* (lung)	6	rs3131043	0.00021	0.00097	rs9468830	0.6728	2.38 × 10^–8^
*LRP4* (spleen)	11	rs2046768	1.43 × 10^–6^	9.85 × 10^–9^	rs7932866	0.9584	2.38 × 10^–9^
*CELF1* (artery tibial)	11	rs7120113	2.10 × 10^–6^	1.63 × 10^–8^	rs12419507	0.0694	4.53 × 10^–9^
*UBALD1* (whole blood)	16	rs3747577	8.63 × 10^–6^	3.17 × 10^–9^	rs12598836	0.4194	2.21 × 10^–10^
*SUGP1* (artery tibial)	19	rs4539728	1.17 × 10^–6^	1.96 × 10^–8^	rs74182632	1	1.43 × 10^–8^
*SUGP1* (pancreas)		rs4539728	1.17 × 10^–6^	1.96 × 10^–8^	rs74182632	1	1.43 × 10^–8^
*TM6SF2* (breast mammary tissue)	19	rs2023883	7.59 × 10^–6^	6.53 × 10^–7^	rs74182632	0.2369	1.43 × 10^–8^
*YJEFN3 *(small intestine terminal)	19	rs8110171	0.000467	0.000412	rs74182632	0.0087	1.43 × 10^–8^

### Co-localisation of putative novel migraine risk genes

Although not near GWS SNP loci in Gormley et al (2016), we examined the putative novel migraine risk genes for co-localisation (COLOC PP4 > 0.5) with SNP association signals (with *P* < 5 × 10^–8^) in Gormley et al (2016). For this purpose, we ran COLOC on all significant gene-tissue pairs, not within ± 500 kb of the 43 autosomal index SNPs from Gormley et al. (2016). At these loci, we identified the number of genes with COLOC PP4 > 0.5 and whether they were at new risk loci identified in Hautakangas et al. (2022). The main purpose of this analysis was to compare each TWAS approach’s ability to identify putative novel risk gene loci. Table 12 shows that although the genes identified by MASHR models had the highest proportion of co-localised genes in new migraine risk loci, the MASHR models identified the lowest total number of co-localised genes at the fewest loci. For example, the best performing MASHR Bonf-Sig-matSpD Top-5 tissue TWAS identified nine genes with PP4 > 0.5 located at four (putative novel) loci. In contrast, the elastic net models identified more co-localised genes at more new migraine loci, with the best performing ENET Bonf-Sig-matSpD Top-5 tissue TWAS, which identified 11 genes with PP4 > 0.5 located at nine (putative novel) loci.

**Table 12:** Co-localisation of putative novel migraine risk genes

Prediction model	Tissues analysed	Multiple test adjustment	No. of genes not within ± 500 kb of the 43 index SNPs (genomic loci)	No. of genes with PP4 > 0.5 (genomic loci)	No. of genes present in new migraine loci (genomic loci)	Proportion of genes in new migraine loci with PP4 > 0.5
ENET	Top 5 tissues	Bonf-Sig	15 (13)	12 (10)	10 (9)	0.83
		Bonf-Sig-matSpD	18 (15)	15 (12)	11 (9)	0.73
	All 49 tissues	Bonf-Sig	17 (9)	10 (5)	8 (4)	0.80
		Bonf-Sig-matSpD	21 (12)	13 (7)	10 (5)	0.77
SMultiXcan	Top 5 tissues	Bonf-Sig	16 (14)	13 (11)	8 (7)	0.62
	All 49 tissues	Bonf-Sig	28 (20)	9 (8)	6 (6)	0.67
MASHR	Top 5 tissues	Bonf-Sig	9 (6)	5 (2)	5 (2)	1
		Bonf-Sig-matSpD	19 (11)	9 (4)	9 (4)	1
	All 49 tissues	Bonf-Sig	6 (5)	1 (1)	1 (1)	1
		Bonf-Sig-matSpD	8 (6)	3 (2)	3 (2)	1

## Discussion

In this paper, migraine GWS loci were characterised using recently developed transcriptome-wide association study (TWAS) approaches. Prior to TWAS approaches, a ‘single eQTL’ approach was used, which tested each GWAS SNP for association with a SNP associated with an eQTL (eSNP). However, this approach has two major drawbacks. Firstly, the multiple testing burden is increased due to testing many eSNPs and secondly, a single eSNP may not completely capture the genetic variation influencing the gene’s expression. The TWAS approach overcomes this hurdle by additively modelling multiple SNPs in the cis-region of the gene, defined as 500 kb upstream of the gene’s transcription start site and 500 kb downstream of the gene’s transcription end site, thus reducing the multiple testing burden and capturing more genetic variation by combining information from multiple SNPs to impute (predict) gene expression. TWAS consists of two steps (i) generation of weights/prediction models for cis-SNPs on gene expression using independent genotype and expression data from healthy individuals, (ii) imputing gene expression values using a reference panel for individual genotype data or GWAS summary statistics and then testing for the association of imputed gene expression values with the trait of interest.

Therefore, the accuracy of gene expression imputation depends upon the quality of prediction models. The early versions of prediction models were elastic net models based upon version 6 and version 7 of GTEx aligned to build 37. The latest models are based on version 8 of GTEx aligned to build 38 and include two classes of prediction models: elastic net and MASHR. The equation used to impute the z-score is the same in both model classes; however, there are differences. Elastic net uses all the cis-SNPs present in the region and generates an additive model to compute SNP weights for gene expression prediction. Elastic net models have a quality check metric that measures the correlation between the predicted transcriptome and assayed expression data (in the reference data such as GTEx). The gene prediction models that have a significant correlation (FDR < 0.05) are made publicly available. This quality check metric is available only for elastic net models. Whereas elastic net uses all cis-SNPs, MASHR only uses fine-mapped SNPs from the DAP-G algorithm to calculate SNP weights for gene expression prediction. MASHR models tend to have a low number of SNPs (mostly 1 or 2 SNPs) incorporated into the prediction model to impute gene expression. All MASHR prediction models are made available for users and there are no model quality checks like the elastic net models.

Both elastic net and MASHR models were used for single-tissue analyses. These analyses showed that the elastic net models were able to identify a greater number of significant differentially expressed genes compared to the MASHR models on already established migraine GWS loci.

GWS loci were characterised based on genes having differential expression and evidence of co-localisation between GWAS risk SNPs and eQTL SNPs. The threshold used for the co-localised signal was PP4 > 0.5 as recommended by the authors. However, an interpretation of these posterior probabilities requires a bit of caution. For example, a gene having low PP4 does not necessarily mean that there is no evidence of co-localisation provided PP3 is low and PP0, PP1 and PP2 are high. This can result from low power in the datasets (Giambartolomei et al. 2014). Therefore, we used two thresholds for the characterisation of a GWAS locus: PP3 < 0.5 and PP4 > 0.5 as an indication of a more robustly co-localised gene.

The differentially expressed and robustly co-localised genes listed in Table 4 have been reviewed elsewhere (Van Den Maagdenberg et al. 2019) and were examined via pathway analysis using the g:GOSt tool (Raudvere et al. 2019). Notably, the genes *ECM1*, *MEF2D*, *PHACTR1*, *FHL5*, *UFL1*, *HEY2*, and *LRP1* were common among all approaches and have all been involved in vascular function. Other genes such as *REST*, *GJA1*, *NCOA7*, *KCNK5*, *PLCE1*, *HTRA1*, *YAP1*, and *ZCCHC14* were not common among all approaches but have also been related to vascular function. Apart from *YAP1*, all of these genes had strong evidence of being co-localised (PP4 > 0.5) with a migraine index SNP. Some of the genes (not common among all approaches) were associated with other plausible pathogenic pathways. For example, *SLC24A3* and *KCNK5* are involved in ion channel activity, and *RNF213* is involved in metal ion homoeostasis. It is interesting given all three genes implicated in a rare monogenic form of migraine—familial hemiplegic migraine (FHM)—are involved in ion transport (Nyholt et al. 2017).

TWAS analyses of the Gormley et al (2016) data also successfully identified significantly differentially expressed genes at putative novel (non-GWS GWAS) loci, that were found to be at new GWS loci in the Hautakangas et al. (2022) GWAS. We also demonstrated that the probability of identifying putative novel risk loci by incorporating eQTL data (as in TWAS) is significantly more than expected by chance. Given the topSNPs of putative novel genes identified in Gormley et al. (2016) had strong evidence for association, they had an increased prior probability of being at a true risk locus and thus have a GWS p value (*P* < 5 × 10^–8^) in Hautakangas et al. (2022). Therefore, we performed a binomial test comparing the proportions of the putative novel migraine risk genes (topSNPs) and topSNPs for all genes. The results show that the TWAS-implicated genes are enriched, whereas genes with similar GWAS significance are not enriched with genome-wide significance in Hautakangas et al. (2022) (binomial test *p* = 2.38 × 10^–20^).

We also compared the different TWAS approaches and multiple testing adjustments in terms of the total number of independent genomic risk loci containing one or more putative migraine risk genes (i.e., index SNPs within 500 kb of each other in Table 10 were merged into the same genomic risk locus). Table 13 shows that compared to the standard Bonferroni adjusted threshold, using the Bonferroni matSpD adjusted threshold (which accounts for the correlation in gene expression within each tissue) identified more putative novel loci for both the top 5 tissue and all 49 tissue analyses. An exception to this was the elastic net model for the top 5 tissues, where the Bonferroni and Bonferroni matSpD threshold identified the same number of putative novel loci.

**Table 13:** Total putative novel genomic risk loci identified by the different prediction models and multiple testing adjustments

Prediction model	Multiple testing adjustment	Top 5 tissues	All 49 tissues
MASHR	Bonferroni	6	4
	Bonferroni matSpD	10	5
ENET	Bonferroni	11	5
	Bonferroni matSpD	11	7
SMultiXcan	Bonferroni	9	14

MASHR models were able to identify 10 and five putative novel genomic risk loci in total for the top 5 tissue and all 49 tissue analyses, respectively. More putative novel genomic risk loci were identified by analysing the top 5 tissues only. The MASHR all 49 tissue analysis identified one locus (near SNP rs56140113) that was not found by the top 5 tissue analysis. The MASHR top 5 tissue analysis identified six loci (near rs68002561/rs7544531, rs72923449, rs7932866, rs12419507, rs28756401, and rs9894634) that were not identified via the all 49 tissue analysis. Four loci (rs12598836, rs74182632, rs1982072, rs28451064) were common between the MASHR all 49 tissue and top 5 tissue analyses.

The elastic net model produced a similar pattern of results. Analysis of the top 5 tissues identified a greater number of putative novel genomic risk loci compared to the analysis of all 49 tissues—identifying 11 and seven loci, respectively. The elastic net top 5 tissue analysis identified seven loci (rs4907224, rs1499963, rs12419507, rs28756401, rs12598836, rs910187/rs3092262, rs28451064) that were not identified via the all 49 tissue analysis. All 49 tissue analysis identified three loci (rs2124663, rs6556059, and rs1982072) that were not found by the top 5 tissue analysis. Four loci (rs1472662, rs68002561/rs7544531, rs74434374, and rs7932866) were common between the elastic net top 5 and all 49 tissue analyses.

The SMultiXcan analysis of all 49 tissues identified a greater number of putative novel genomic risk loci compared to the analysis of the top 5 tissues—identifying 14 and nine loci, respectively. Two loci (rs1499963 and rs28756401) were specific to the top 5 tissues analysis. Seven loci (rs2124663, rs12057629/rs61561984, rs4907224, rs6556059, rs4285, rs9468830, and rs74182632) were exclusive to all 49 tissues analysis. Seven loci (rs1472662, rs68002561/rs7544531, rs56140113, rs74434374, rs7932866, rs12598836, and rs28451064) were identified via both the SMultiXcan top 5 and all 49 tissue analyses.

Table 11 shows that our pipeline was able to identify nine putative novel migraine risk genes significantly differentially expressed in TWAS analysis of Gormley et al. (2016) data. It is interesting to note that some of these genes were expressed in the tissues that were not directly related to migraine, for example, breast mammary tissue. Genes significantly differentially expressed in such tissues could result from individual or combinations of factors such as GTEx tissue sample sizes, gene co-regulation/co-expression and/or isoform abundance of pathogenic genes in a particular tissue (Ghaffar and Nyholt 2022). However, these genes were later found to be at (and in LD with) true (GWS) migraine risk index SNP loci in Hautakangas et al (2022). Six of these genes (*RP11-353N4.6*, *EIF2D*, *LRP4*, *UBALDI*, *SUGP1*, and *TM6SF2*) are at loci with multiple differentially expressed genes, some of which were implicated by MetaXcan and/or FOCUS analysis by Hautakangas et al. (2022). However, there were three genes *ABHD16A* (near rs74434374), *HCG20* (near rs9468830), and *HOXD8* (near rs72923449) identified by our TWAS pipeline analysis that were not implicated by MetaXcan or FOCUS analysis by Hautakangas et al. (2022). *ABHD16A* is a member of the alpha/beta hydrolase domain-containing protein family that is involved in Kawasaki disease (Xu et al. 2018). Kawasaki disease is a disease caused by inflammation of blood vessels, thus having a vascular component. *HCG20* is a non-coding RNA gene associated with brain malformations and major depressive disorder (Li et al. 2019). *HOXD8* belongs to a homeobox family of genes and has a tumour suppressing role in different cancers by inducing apoptosis and inhibiting proliferation (Zhang et al. 2021). Hypermethylation of *HOXD8* is used as a biomarker to detect biliary tract cancers (Loi et al. 2022). *ABHD16A* can directly be associated with migraine because of its potential vascular role. *HCG20* is associated with major depressive disorder, that in turn is correlated with migraine (Yang et al. 2018). *HOXD8* is a gene playing important role in different cancers but its involvement in migraine aetiology is unclear.

TWAS approaches can be viewed as methods directed to improve the discovery power of the GWAS without increasing sample size by incorporating functional information. Some authors suggest that the secondary usage of these methods is to increase the statistical power of GWAS to identify novel loci (Moore et al. 2022). We have shown in our analysis that the number of putative novel loci identified using Gormley et al. (2016) migraine GWAS that were GWS in the latest Hautakangas et al. (2022) migraine GWAS is significantly more than expected by chance (one-sided binomial test *p* = 2.38 × 10^–20^). Thus, performing TWAS analyses on the latest more powerful migraine GWAS would be expected to identify additional novel migraine risk loci.

The migraine GWAS performed by Gormley et al. (2016) and Hautakangas et al. (2022) were in populations of European ancestry and the TWAS prediction models from GTEx were derived from samples of predominantly (84.6%) white populations. Most migraine GWAS have been performed in individuals of European descent, hence the results may not be directly transferable to other ancestral populations. There have been studies that tried to replicate the European risk loci in relatively small non-European populations. One of the studies in a Chinese population replicated one of the migraine risk loci from the three risk variants known at that time (An et al. 2013; Fan et al. 2014). A subsequent Chinese replication study of 581 migraine cases and 533 ethnically matched controls identified three risk loci rs2274316 (*MEF2D*), rs6478241 (*ASTN2*) and rs2651899 (*PRDM16*) previously identified in European samples (An et al. 2017). A later study in a North Indian population replicated three loci; rs1835740 (near *MTDH*), rs11172113 (*LRP1*), rs2651899 (*PRDM16*) (Ghosh et al. 2013). In addition to replicating European risk loci, a few migraine GWAS have been performed in Asian populations but all had small sample sizes (Jiang et al. 2021; Tsai et al. 2021; Tsao et al. 2022). Thus, it is reasonable to conclude that migraine GWAS in European populations have produced many migraine risk variants, and despite a lack of replication power, some of these loci have been replicated in non-European populations (Harder et al. 2023). Regardless, as for the vast majority of GWAS traits performed to date, additional and larger migraine GWAS in non-European populations is required to identify additional and perhaps ancestry-specific risk loci. Similarly, although studies comparing TWAS using European and non-European transcriptome prediction models found that z-scores for differential expression are highly correlated (e.g., Pearson correlation of 0.63 between an African American and Hispanic/Latino model and a European model) (Geoffroy et al. 2020), given allele frequencies and effect sizes can differ across ancestral populations (Mogil et al. 2018), TWAS with population-matched transcriptome models should have more power to identify trait-associated and colocalised genes and thus more transcriptome studies in diverse populations are needed.

Overall, our TWAS analyses of the Gormley et al (2016) migraine GWAS identified 21 independent putative novel risk loci harbouring putative migraine risk genes. Universally, compared to the MASHR models, the elastic net models identified more putative novel risk genes and loci, and SMultiXcan analysis of all 49 tissues identified the most putative novel risk loci shown to be true risk loci in the recent more powerful migraine GWAS by Hautakangas et al. (2022).

## Supplementary Information

Below is the link to the electronic supplementary material.

Supplementary file1 (XLSX 16 KB)

Supplementary file2 (XLSX 21 KB)

Supplementary file3 (XLSX 43 KB)

Supplementary file4 (XLSX 24 KB)

Supplementary file5 (XLSX 60 KB)

Supplementary file6 (DOCX 44 KB)

## Data Availability

The migraine GWAS summary statistics are obtained from the 23andMe and IHGC. The full GWAS summary statistics for the 23andMe discovery data set will be made available through 23andMe to qualified researchers under an agreement with 23andMe that protects the privacy of the 23andMe participants. For further details and to access the data, please visit https://research.23andme.com/collaborate/#dataset-access/. Gene expression and eQTL data are freely available at https://gtexportal.org/home/.
