# Supplementary material for: Integrating eQTL and GWAS data characterises established and identifies novel migraine risk loci
Source: Hum Genet. 2023 May 28;142(8):1113–37. doi: 10.1007/s00439-023-02568-8 (PMC10449685; doi:10.1007/s00439-023-02568-8)
Supplement: Supplementary file 6 — Supplementary file6 (DOCX 44 KB) [file 439_2023_2568_MOESM6_ESM.docx]

**Supplementary Table 6** Genes crossing Bonferroni and Bonferroni matSpD thresholds for all 49 tissues using elastic net models. GWAS p-value of predictor SNP for each gene in Gormley 2016 and Hautakangas 2022 migraine GWASs is listed. LD between predictor SNP and lead variant from Hautakangas 2022 is shown. Underlined genes are found exclusively with Bonferroni matSpD threshold.

| Gene (Tissue) | Chr | Predictor SNP | GWAS P-value for predictor SNP in  Gormley et al. (2016) | GWAS P-value for predictor SNP in Hautakangas et al. (2022) | Lead index SNP in Hautakangas et al. (2022) | LD between predictor and lead index SNP | GWAS P-value for lead SNP in Hautakangas et al. (2022) |
| --- | --- | --- | --- | --- | --- | --- | --- |
| *MACF1* (Thyroid)  *BMP8A* (Artery Aorta)  *BMP8A* (Artery Tibial)  *BMP8A* (Esophagus gastroesophageal Junction)  *BMP8A* (Heart Left ventricle)  *PABPC4* (Lung)  *PABPC4* (Colon Transverse) | 1 | rs4660846  rs710913  rs710913  rs710913  rs710913  rs7539261  rs3738676 | 7.28E-07  7.00E-07  7.00E-07  7.00E-07  7.00E-07  7.59E-07  3.68E-07 | 3.21E-07  2.32E-07  2.32E-07  2.32E-07  2.32E-07  3.27E-07  1.02E-07 | rs1472662 | 0.278  0.2743  0.2743  0.2743  0.2743  0.2809  0.2966 | 1.75E-08 |
| *TNFRSF14* (Small Intestine Terminal) | 1 | rs868718 | 3.89E-17 | 1.57E-36 | rs2124663 | 0.0003 | 2.44E-09 |
| *HIST2H2BF* (Esophagus Muscularis)  *BOLA1* (Adipose Visceral Omentum)  *SV2A* (Artery Aorta)  *SV2A* (Colon Transverse)  *SV2A* (Esophagus Gastroesophageal Junction)  *SV2A* (Lung)  *SV2A* (Artery Coronary)  *SV2A* (Esophagus Muscularis)  *SV2A* (Nerve Tibial) | 1 | rs698915  rs6693567  rs17643644  rs10494363  rs12078573  rs10494363  rs12078573  rs12078573  rs10494363 | 4.62E-07  1.21E-08  3.84E-07  1.96E-07  2.78E-07  1.96E-07  2.78E-07  2.78E-07  1.96E-07 | 3.47E-11  1.25E-13  6.20E-09  3.01E-08  6.91E-09  3.01E-08  6.91E-09  6.91E-09  3.01E-08 | rs68002561 | 0.0004  0.0  0.8199  0.7860  0.8086  0.7860  0.8086  0.8086  0.7860 | 3.61E-10 |
| *NKX2-5* (Skin Exposed Lower Leg) | 5 | rs9313619 | 8.72E-08 | 2.44E-09 | rs6556059 | 0.9155 | 8.15E-10 |
| *ABHD16A* (Pancreas) | 6 | rs1802127 | 6.25E-06 | 4.16E-06 | rs74434374 | 0.1828 | 4.51E-09 |
| *CYP2C9* (Skin Exposed Lower Leg) | 10 | rs7075182 | 3.61E-07 | 1.15E-11 | rs2274224 | 0.2464 | 3.29E-26 |
| *MDK* (Esophagus Muscularis)  *ATG13* (testis)  *ATG13* (Nerve Tibial)  *LRP4* (Spleen) | 11 | rs12574668  rs12574668  rs12574668  rs2046768 | 6.02E-07  6.02E-07  6.02E-07  1.43E-06 | 5.34E-09  5.34E-09  5.34E-09  9.85E-09 | rs7932866 | 0.9521  0.9521  0.9521  0.9584 | 2.38E-09 |
| *EXOSC5* (Adrenal Gland)  *B9D2* (Testis) | 19 | rs11666933  rs1800469 | 5.49E-07  1.46E-07 | 1.32E-09  5.13E-11 | rs1982072 | 0.7016  0.9954 | 4.22E-11 |

**Supplementary Table 7** Genes crossing Bonferroni and Bonferroni matSpD thresholds for top 5 tissues using elastic net models. GWAS p-value of predictor SNP for each gene in Gormley 2016 and Hautakangas 2022 migraine GWASs is listed. LD between predictor SNP and lead variant from Hautakangas 2021 is shown. Underlined genes are found exclusively with Bonferroni matSpD threshold.

| Gene (Tissue) | Chr | Predictor SNP | GWAS P-value for predictor SNP in  Gormley et al. (2016) | GWAS P-value for predictor SNP in Hautakangas et al. (2022) | Lead index SNP in Hautakangas et al. (2022) | LD between predictor and lead index SNP | GWAS P-value for lead SNP in Hautakangas et al. (2022) |
| --- | --- | --- | --- | --- | --- | --- | --- |
| *BMP8A* (Artery Aorta)  *BMP8A* (Artery Tibial)  *PABPC4* (Artery Tibial) | 1 | rs710913  rs710913  rs1775654 | 7.00E-07  7.00E-07  1.33E-06 | 2.32E-07  2.32E-07  4.66E-07 | rs1472662 | 0.2743  0.2743  0.5567 | 1.75E-08 |
| *SV2A* (Artery Aorta)  *SV2A* (Artery coronary) | 1 | rs17643644  rs12078573 | 3.84E-07  2.78E-07 | 6.20E-09  6.91E-09 | rs68002561 | 0.8199  0.8086 | 3.61E-10  1.31E-10 |
| *TMEM127*(Artery Tibial) | 2 | rs4426565 | 8.64E-07 | 4.09E-08 | rs4907224 | 0.5127 | 1.63E-09 |
| *ITGB5* (Artery coronary) | 3 | rs11928651 | 5.65E-06 | 1.46E-07 | rs1499963 | 0.3358 | 7.48E-09 |
| *ABHD16A* (Pancreas) | 6 | rs1802127 | 6.25E-06 | 4.16E-06 | rs74434374 | 0.1828 | 4.51E-09 |
| *MDK* (Artery Tibial)  *LRP4* (spleen) | 11 | rs7481312  rs2046768 | 7.15E-07  1.43E-06 | 1.02E-08  9.85E-09 | rs7932866 | 0.986  0.9584 | 2.38E-09 |
| *CELF1* (Artery Tibial) | 11 | rs7120113 | 2.10E-06 | 1.63E-08 | rs12419507 | 0.0694 | 4.53E-09 |
| *LINC00216* (Pancreas) | 14 | rs7157304 | 7.46E-07 | 4.16E-08 | rs28756401 | 0.8844 | 6.41E-09 |
| *HMOX2* (Artery Tibial)  *HMOX2* (Spleen) | 16 | rs4786490  rs4786490 | 5.40E-07  5.40E-07 | 1.81E-08  1.81E-08 | rs12598836 | 0.7349  0.7349 | 2.21E-10 |
| *EYA2* (Artery Tibial) | 20 | rs1212593 | 3.03E-06 | 5.83E-07 | rs910187  rs3092262 | 0.0015  0.7734 | 1.14E-10  1.08E-08 |
| *LINC00310* (Artery Aorta) | 21 | rs9305545 | 1.16E-06 | 3.90E-12 | rs28451064 | 0.8197 | 3.52E-15 |

**Supplementary Table** **8** Genes crossing Bonferroni threshold for all 49 tissues using SMultiXcan. GWAS p-value of predictor SNP for each gene in Gormley 2016 and Hautakangas 2022 migraine GWASs is listed. LD between predictor SNP and lead variant from Hautakangas 2022 is shown.

| Gene (Tissue) | Chr | Predictor SNP | GWAS P-value for predictor SNP in  Gormley et al. (2016) | GWAS P-value for predictor SNP in Hautakangas et al. (2022) | Lead index SNP in Hautakangas et al. (2022) | LD between predictor and lead index SNP | GWAS P-value for lead SNP in Hautakangas et al. (2022) |
| --- | --- | --- | --- | --- | --- | --- | --- |
| *TMEM51* (Esophagus Mucosa) | 1 | rs10737909 | 1.04E-07 | 3.52E-12 | rs12057629  rs61561984 | 0.5067  0.1227 | 9.38E-14  1.59E-08 |
| *MACF1*(Thyroid) | 1 | rs4660846 | 7.28E-07 | 3.21E-07 | rs1472662 | 0.278 | 1.75E-08 |
| *EIF2D* (Thyroid) | 1 | rs4072677 | 1.06E-05 | 5.83E-06 | rs56140113 | 0.2291 | 7.76E-09 |
| *TNFRSF14* (Small Intestine Terminal) | 1 | rs868718 | 3.89E-17 | 1.57E-36 | rs2124663 | 0.0003 | 2.44E-09 |
| *HIST2H2BF* (Esophagus Muscularis)  *BOLA1* (Adipose Visceral Omentum)  *RP11-353N4.6* (Lung)  *FCGR1A* (Whole Blood) | 1 | rs698915  rs6693567  rs1046332  rs698915 | 4.62E-07  1.21E-08  1.76E-06  4.62E-07 | 3.47E-11  1.25E-13  1.31E-07  3.47E-11 | rs68002561 | 0.0004  0.0  0.7383  0.0004 | 3.61E-10 |
| *ITPRIPL1* (Small Intestine Terminal) | 2 | rs4426565 | 8.64E-07 | 4.09E-08 | rs4907224 | 0.5127 | 1.63E-09 |
| *POC5* (Brain cortex) | 5 | rs2047059 | 1.70E-06 | 1.90E-10 | rs42854 | 0.5982 | 9.39E-13 |
| *NKX2-5* (Skin Exposed Lower Leg) | 5 | rs9313619 | 8.72E-08 | 2.44E-09 | rs6556059 | 0.9155 | 8.15E-10 |
| *HCG20* (Lung) | 6 |  |  |  | rs9468830 |  | 2.38E-08 |
| *ABHD16A* (Pancreas) | 6 | rs1802127 | 6.25E-06 | 4.16E-06 | rs74434374 | 0.1828 | 4.51E-09 |
| *CYP2C9* (Skin Exposed Lower Leg) | 10 | rs7075182 | 3.61E-07 | 1.15E-11 | rs2274224 | 0.2464 | 3.29E-26 |
| *LRP4* (Spleen) | 11 | rs2046768 | 1.43E-06 | 9.85E-09 | rs7932866 | 0.9584 | 2.38E-09 |
| *NMRAL1* (Heart left ventricle)  *UBALD1* (Whole Blood) | 16 | rs4786490  rs3747577 | 5.40E-07  8.63E-06 | 1.81E-08  3.17E-09 | rs12598836 | 0.7349  0.4194 | 2.21E-10 |
| *TM6SF2* (breast mammary tissue)  *GATAD2A* (Skin not exposed suprapubic)  *YJEFN3 (*Small Intestine Terminal*)* | 19 | rs2023883  rs2074301  rs8110171 | 7.59E-06  1.85E-05  0.000467 | 6.53E-07  1.96E-06  0.000412 | rs74182632 | 0.2369  0.2406  0.0087 | 1.43E-08 |
| *LINC00310* (Artery Aorta) | 21 | rs9305545 | 1.16E-06 | 3.90E-12 | rs28451064 | 0.8197 | 3.52E-15 |

**Supplementary Table 9** Genes crossing Bonferroni threshold for top 5 tissues using SMultiXcan. GWAS p-value of predictor SNP for each gene in Gormley 2016 and Hautakangas 2022 migraine GWASs is listed. LD between predictor SNP and lead variant from Hautakangas 2022 is shown.

| Gene (Tissue) | Chr | Predictor SNP | GWAS P-value for predictor SNP in  Gormley et al. (2016) | GWAS P-value for predictor SNP in Hautakangas et al. (2022) | Lead index SNP in Hautakangas et al. (2022) | LD between predictor and lead index SNP | GWAS P-value for lead SNP in Hautakangas et al. (2022) |
| --- | --- | --- | --- | --- | --- | --- | --- |
| *BMP8A* (Artery Aorta)  *PABPC4 (Artery Tibial)* | 1 | rs710913  rs1775654 | 7.00E-07  1.33E-06 | 2.32E-07  4.66E-07 | rs1472662 | 0.2743  0.5567 | 1.75E-08 |
| *EIF2D (Spleen)* | 1 | rs6658181 | 1.45E-05 | 7.49E-05 | rs56140113 | 0.0159 | 7.76E-09 |
| *SV2A (Artery Aorta)* | 1 | rs17643644 | 3.84E-07 | 6.20E-09 | rs68002561 | 0.8199 | 3.61E-10 |
| *ITGB5 (Artery coronary)* | 3 | rs11928651 | 5.65E-06 | 1.46E-07 | rs1499963 | 0.3358 | 7.48E-09 |
| *ABHD16A (Pancreas)* | 6 | rs1802127 | 6.25E-06 | 4.16E-06 | rs74434374 | 0.1828 | 4.51E-09 |
| *LRP4 (Spleen)* | 11 | rs2046768 | 1.43E-06 | 9.85E-09 | rs7932866 | 0.9584 | 2.38E-09 |
| *LINC00216* (Pancreas) | 14 | rs7157304 | 7.46E-07 | 4.16E-08 | rs28756401 | 0.8844 | 6.41E-09 |
| *HMOX2 (Spleen)* | 16 | rs4786490 | 5.40E-07 | 1.81E-08 | rs12598836 | 0.7349 | 2.21E-10 |
| *LINC00310 (Artery Aorta)* | 21 | rs9305545 | 1.16E-06 | 3.90E-12 | rs28451064 | 0.8197 | 3.52E-15 |

**Supplementary Table 10** Genes crossing Bonferroni and Bonferroni matSpD thresholds for all 49 tissues using MASHR models. GWAS p-value of predictor SNP for each gene in Gormley 2016 and Hautakangas 2022 migraine GWASs is listed. LD between predictor SNP and lead variant from Hautakangas 2022 is shown. Underlined genes are found exclusively with Bonferroni matSpD threshold.

| Gene (Tissue) | Chr | Predictor SNP | GWAS P-value for predictor SNP in  Gormley et al. (2016) | GWAS P-value for predictor SNP in Hautakangas et al. (2022) | Lead index SNP in Hautakangas et al. (2022) | LD between predictor and lead index SNP | GWAS P-value for lead SNP in Hautakangas et al. (2022) |
| --- | --- | --- | --- | --- | --- | --- | --- |
| *MAPKAPK2* (Esophagus Mucosa) | 1 | rs3935564 | 3.05E-07 | 1.45E-08 | rs56140113 | 0.9014 | 7.76E-09 |
| *DNAJA3* (Brain Hippocampus)  *DNAJA3* (Brain Putamen Basal Ganglia)  *DNAJA3* (Kidney Cortex)  *DNAJA3* (Brain Caudate Basal Ganglia)  *DNAJA3* (Brain Substantia Nigra)  *HMOX2* (Nerve Tibial)  *HMOX2* (Prostrate)  *HMOX2* (Adipose Visceral Omentum)  *HMOX2* (Artery Aorta)  *HMOX2* (Artery Tibial)  *HMOX2* (Breast Mammary Tissue)  *HMOX2* (Esophagus Gastrophageal Junction)  *HMOX2* (Esophagus Mucosa)  *HMOX2* (Lung)  *HMOX2* (Thyroid)  *NMRAL1* (Thyroid) | 16 | rs17137018  rs17137018  rs17137018  rs17137018  rs17137018  rs4785967  rs3761680  rs4785967  rs4785967  rs4786501  rs4785967  rs4785967  rs4785967  rs4785967  rs4785967  rs7194761 | 1.27E-04  1.27E-04  1.27E-04  1.27E-04  1.27E-04  1.17E-07  1.23E-06  1.17E-07  1.17E-07  1.81E-07  1.17E-07  1.17E-07  1.17E-07  1.17E-07  1.17E-07  9.07E-07 | 5.85E-04  5.85E-04  5.85E-04  5.85E-04  5.85E-04  1.93E-09  1.58E-08  1.93E-09  1.93E-09  3.38E-09  1.93E-09  1.93E-09  1.93E-09  1.93E-09  1.93E-09  1.68E-08 | rs12598836 | 0.1095  0.1095  0.1095  0.1095  0.1095  1  0.9472  1  1  1  1  1  1  1  1  0.943 | 2.21E-10 |
| *MAU2* (Esophagus Muscularis) | 19 | rs4539728 | 1.17E-06 | 1.96E-08 | rs74182632 | 1 | 1.43E-08 |
| *B9D2* (Prostate) | 19 | rs2317131 | 1.08E-06 | 1.82E-07 | rs1982072 | 0.2565 | 4.22E-11 |
| *KCNE2* (Artery Aorta) | 21 | rs60687229 | 1.41E-07 | 5.43E-12 | rs28451064 | 0.6738 | 3.52E-15 |

**Supplementary Table 11** Genes crossing Bonferroni and Bonferroni matSpD thresholds for top 5 tissues using MASHR models. GWAS p-value of predictor SNP for each gene in Gormley 2016 and Hautakangas 2022 migraine GWASs is listed. LD between predictor SNP and lead variant from Hautakangas 2022 is shown. Underlined genes are found exclusively with Bonferroni matSpD threshold.

| Gene (Tissue) | Chr | Predictor SNP | GWAS P-value for predictor SNP in  Gormley et al. (2016) | GWAS P-value for predictor SNP in Hautakangas et al. (2022) | Lead index SNP in Hautakangas et al. (2022) | LD between predictor and lead index SNP | GWAS P-value for lead SNP in Hautakangas et al. (2022) |
| --- | --- | --- | --- | --- | --- | --- | --- |
| *SF3B4* (Artery Coronary)  *SF3B4* (Pancreas*)*  *SV2A* (Artery Aorta)  *SV2A* (Artery Coronary)  *SV2A* (Artery Tibial)  *SV2A* (Pancreas)  *SV2A* (Spleen) | 1 | rs17643644  rs17643644  rs112851681  rs112851681  rs112851681  rs112851681  rs112851681 | 3.84E-07  3.84E-07  9.77E-07  9.77E-07  9.77E-07  9.77E-07  9.77E-07 | 6.20E-09  6.20E-09  2.66E-08  2.66E-08  2.66E-08  2.66E-08  2.66E-08 | rs68002561 | 0.8199  0.8199  0.8513  0.8513  0.8513  0.8513  0.8513 | 3.61E-10 |
| *HOXD8* | 2 | rs114763776 | 1.97E-05 | 0.062848 | rs72923449 | 0.2979 | 4.66E-08 |
| *CHRM4* (Artery Aorta)  *HARBI1*(Artery Aorta)  *HARBI1*(Artery Coronary)  *HARBI1*(Artery Tibial)  *HARBI1*(Pancreas)  *HARBI1*(Spleen)  *ATG13*(Pancreas)  *ATG13*(Spleen) | 11 | rs2067482  rs61884264  rs61884264  rs61884264  rs61884264  rs61884264  rs61884264  rs61884264 | 7.07E-07  1.00E-06  1.00E-06  1.00E-06  1.00E-06  1.00E-06  1.00E-06  1.00E-06 | 9.99E-09  9.76E-09  9.76E-09  9.76E-09  9.76E-09  9.76E-09  9.76E-09  9.76E-09 | rs7932866 | 0.9789  0.9587  0.9587  0.9587  0.9587  0.9587  0.9587  0.9587 | 2.38E-09 |
| *FNBP4* (spleen) | 11 | rs12223593 | 6.00E-06 | 5.23E-07 | rs12419507 | 0.2685 | 4.54E-09 |
| *ARID4A* (Spleen) | 14 | rs1957038 | 1.22E-06 | 7.43E-09 | rs28756401 | 0.9949 | 6.41E-09 |
| *HMOX2* (Artery Aorta)  *HMOX2* (Artery Tibial)  *HMOX2* (Spleen) | 16 | rs4785967  rs4786499  rs4786490 | 1.17E-07  2.68E-04  5.40E-07 | 1.93E-09  5.99E-04  1.81E-08 | rs12598836 | 1  0.2623  0.7349 | 2.21E-10 |
| *RP11-667K14.3* (Artery Coronary) | 17 | rs7216804 | 9.77E-07 | 1.88E-07 | rs9894634 | 0.4051 | 9.63E-11 |
| *MAU2* (Artery Coronary)  *MAU2* (Artery Aorta)  *SUGP1*(Artery Tibial)  *SUGP1*(Pancreas) | 19 | rs4539728  rs4539728  rs4539728  rs4539728 | 1.17E-06  1.17E-06  1.17E-06  1.17E-06 | 1.96E-08  1.96E-08  1.96E-08  1.96E-08 | rs74182632 | 1  1  1  1 | 1.43E-08 |
| *B9D2*(Artery Coronary) | 19 | rs2241712 | 2.29E-06 | 2.30E-10 | rs1982072 | 0.9378 | 4.22E-11 |
| *MRPS6* (Artery Aorta)  *SLC5A3* (Artery Aorta)  *AP000318.2* (Artery Aorta)  *KCNE2* (Artery Aorta)  *LINC00310* (Artery Aorta) | 21 | rs28451064  rs28451064  rs28451064  rs60687229  rs9305545 | 2.69E-07  2.69E-07  2.69E-07  1.41E-07  1.16E-06 | 3.52E-15  3.52E-15  3.52E-15  5.43E-12  3.90E-12 | rs28451064 | 1  1  1  0.6738  0.8197 | 3.52E-15 |
